# A Dual Function for Prickle in Regulating Frizzled Stability during Feedback-Dependent Amplification of Planar Polarity

**DOI:** 10.1016/j.cub.2017.08.016

**Published:** 2017-09-25

**Authors:** Samantha J. Warrington, Helen Strutt, Katherine H. Fisher, David Strutt

**Affiliations:** 1 Bateson Centre, Firth Court, University of Sheffield, Sheffield S10 2TN, UK; 2 Department of Biomedical Science, Firth Court, University of Sheffield, Sheffield S10 2TN, UK

**Keywords:** planar polarity, planar cell polarity, PCP, Frizzled, *Drosophila*, self-organization, feedback

## Abstract

The core planar polarity pathway coordinates epithelial cell polarity during animal development, and loss of its activity gives rise to a range of defects, from aberrant morphogenetic cell movements to failure to correctly orient structures, such as hairs and cilia. The core pathway functions via a mechanism involving segregation of its protein components to opposite cells ends, where they form asymmetric intracellular complexes that couple cell-cell polarity. This segregation is a self-organizing process driven by feedback interactions between the core proteins themselves. Despite intense efforts, the molecular pathways underlying feedback have proven difficult to elucidate using conventional genetic approaches. Here we investigate core protein function during planar polarization of the *Drosophila* wing by combining quantitative measurements of protein dynamics with loss-of-function genetics, mosaic analysis, and temporal control of gene expression. Focusing on the key core protein Frizzled, we show that its stable junctional localization is promoted by the core proteins Strabismus, Dishevelled, Prickle, and Diego. In particular, we show that the stabilizing function of Prickle on Frizzled requires Prickle activity in neighboring cells. Conversely, Prickle in the same cell has a destabilizing effect on Frizzled. This destabilizing activity is dependent on the presence of Dishevelled and blocked in the absence of Dynamin and Rab5 activity, suggesting an endocytic mechanism. Overall, our approach reveals for the first time essential in vivo stabilizing and destabilizing interactions of the core proteins required for self-organization of planar polarity.

## Highlights

• Dishevelled, Strabismus, and Prickle cooperate to stabilize planar polarity complexes
• Prickle acts via Strabismus to stabilize Frizzled in neighboring cells
• Prickle acts via Dishevelled to destabilize Frizzled in the same cell
• Frizzled destabilization by Prickle depends on active endocytosis

## Graphical Abstract

**Figure:**
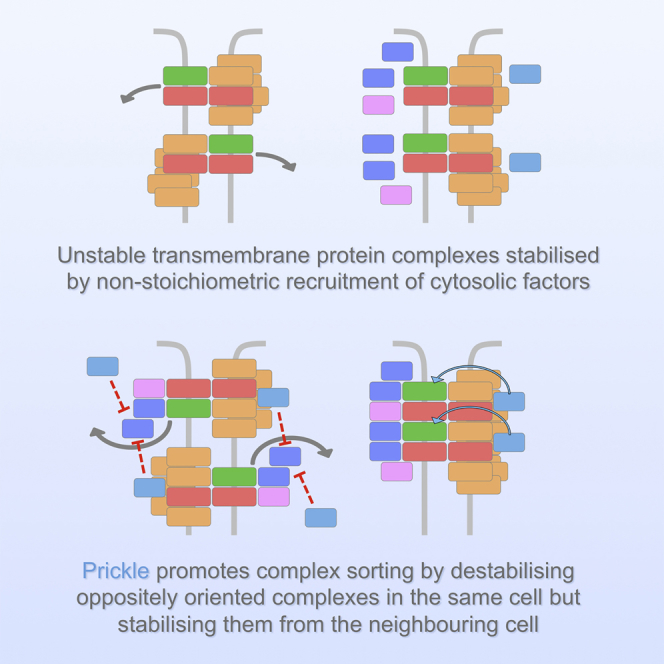

## Teaser

Warrington et al. use quantitative measurement of protein dynamics together with precise spatiotemporal control of gene function to dissect activities of the core planar polarity pathway. This reveals an unexpected dual function of the core pathway protein Prickle, in both stabilizing core protein complexes in *trans* and destabilizing them in *cis*.

## Introduction

Planar polarization describes the property whereby cells show coordinated polarized behaviors within the plane of a tissue and underlies diverse phenomena, including hairs, bristles, and cilia adopting a common orientation on the surface of an epithelium, and groups of cells or neurons showing coordinated directional migrations [1, 2]. Throughout the animal kingdom, the major molecular pathway controlling planar polarity is the so-called “core” pathway [3].

**Figure 1:**
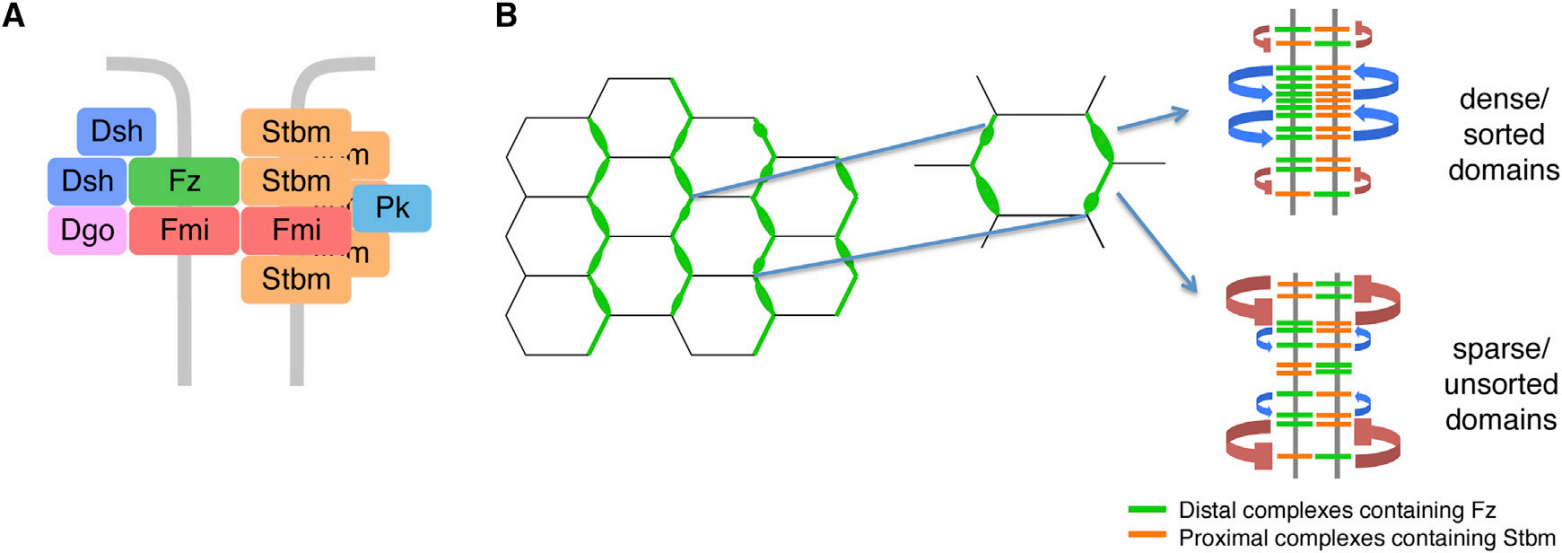
The Core Planar Polarity Protein Complex and Feedback Amplification of Asymmetry (A) The core proteins form non-stoichiometric asymmetric intercellular complexes at cell junctions around a trans-dimer of Fmi (red) bound to Fz (green) on one side of the junction and Stbm (orange) on the other, associated with the cytoplasmic proteins Dsh (dark blue), Pk (light blue), and Dgo (pink). (B) During normal development, the asymmetric core protein complexes adopt polarized distributions within cells, such that Fz, Dsh, and Dgo predominantly localize to distal cell edges (right side of cells in diagram; only Fz [green] shown) and Stbm and Pk to proximal cells edges (left side of cells in diagram; only Stbm [orange] shown), with Fmi at both cell edges. At the cell edges, the proteins are concentrated in bright regions (“puncta”), where most complexes are in the same orientation (inset at top right), interspersed with less bright regions where complexes of mixed orientations are found (inset at bottom right). Amplification of asymmetry and concentration in puncta are believed to be driven by either positive (stabilizing) interactions between complexes of like orientation (blue arrows) or negative (destabilizing) interactions between complexes of unlike orientation (red flat-headed arrows). Stabilizing interactions will predominate in sorted domains and destabilizing interactions in unsorted domains. Proximodistal orientation of the complexes is believed to be driven by a variety of global cues.

Based primarily on studies in the developing *Drosophila* wing, it has been found that the core pathway proteins have the ability to assemble into asymmetric intercellular junctional complexes formed around a backbone of an intercellular homodimer of the cadherin Flamingo (Fmi; also known as Starry Night) associated on one side of the junction with the seven-pass transmembrane protein Frizzled (Fz). The other components are the four-pass transmembrane protein Strabismus (Stbm; also known as Van Gogh), which associates with Fmi on the opposite side of the junction to Fz, the cytoplasmic proteins Dishevelled (Dsh) and Diego (Dgo) that colocalize with Fz, and the cytoplasmic protein Prickle (Pk) that colocalizes with Stbm [4]. Interestingly, whereas Fmi and Fz appear to associate stoichiometrically in a 2:1 ratio, the other components of the complex show variable stoichiometries, suggesting a “signalosome-like” organization (Figure 1A) [5]. Such an organization might serve to provide complexes with intrinsic stability, as a result of weak multivalent interactions between complex components driving a phase transition into a stable state [6].

In planar polarized tissues, the core proteins are sorted within cells such that Fz, Dsh, and Dgo lie at one end of the cell and Stbm and Pk lie at the other. These subcellular distributions then act as polarity cues within the cell, interacting with downstream “effector” pathways to mediate polarized cell behaviors [7, 8]. It is generally believed that the overall direction of polarization in a tissue is determined by gradients and boundaries of morphogen expression that define the tissue axes [9, 10]. These are proposed to specify biases in core protein activity across cell axes, which are then amplified by feedback mechanisms.

The most striking evidence for feedback amplification comes from the observations that groups of cells lacking the core proteins Fz and Stbm are able to reorganize the polarity of neighboring cells [11, 12, 13, 14, 15, 16]. This shows that cells can adopt strongly polarized states independently of tissue-level cues and that cell polarity is intrinsically coupled between neighboring cells.

To understand the feedback mechanisms, a number of theoretical/computational models for planar polarization by the core pathway have been presented (e.g., [17, 18, 19, 20, 21, 22]), which share two common features: (1) the core proteins form asymmetric intercellular complexes at cell junctions, with Fz and its binding partners on one side of the cell junction and Stbm and its partners on the other, and (2) feedback interactions occur between the core proteins of either a positive nature such that “like” complexes of the same orientation are stabilized or of a negative nature such that “unlike” complexes of opposite orientation are destabilized (Figure 1B).

Notably, core protein localization at cell junctions is concentrated in puncta in which intercellular complexes share a common orientation [23, 24], and core proteins concentrated in such clusters show lower turnover than core proteins in other regions of the junctions [5, 23]. Taken together with the theoretical studies, these results suggest that feedback interactions occur locally between core protein complexes to produce individual domains of stable sorted complexes (Figure 1B).

Most attempts to identify molecular mechanisms of feedback have centered on overexpression assays, which have provided evidence for both stabilizing and destabilizing interactions between the core proteins. For instance, overexpression of the cytoplasmic core proteins Dsh, Pk, or Dgo causes formation of large punctate accumulations of the core proteins at cell junctions [16, 25, 26], consistent with these factors promoting stable clustering of complexes. Furthermore, it has been demonstrated in a heterologous cell culture assay that overexpression of Pk or Stbm can cause displacement of Dsh from Fz-Dsh membrane complexes [18, 26], most likely due to competitive binding. Similarly competitive binding interactions between the core proteins Dgo, Pk, and Dsh have been shown in both in vitro binding assays and in vivo overexpression assays [27]. More recently, it has been reported that in vivo overexpression of Pk can trigger the appearance of large intracellular vesicles containing Pk, Fmi, and Stbm [24], providing evidence for Pk activity mediating the removal of Stbm-containing complexes. These observations are all consistent with destabilizing feedback interactions between Fz and Stbm complexes.

However, there are caveats regarding the conclusions of these overexpression studies. First, they rely on non-physiological levels of expression. Second, mechanisms relying on competitive mass action binding reactions might exhibit rather slow kinetics given that the relative concentrations of core proteins in the junctions are all very similar [5]: a more plausible mechanism would involve some form of catalysis with more rapid kinetics.

One such potential feedback interaction, identified in mouse, relies on the catalytic mechanism of ubiquitination. Ubiquitin ligases of the Smurf family can regulate levels of a vertebrate Pk homolog (Pk1), and in tissue culture, Smurf-mediated degradation of Pk1 is promoted by association of Smurf with the vertebrate Dsh homolog Dvl2 [28]. However, this pathway does not appear to exist in *Drosophila* [24, 29].

Overall, understanding the molecular mechanisms of planar polarity remains a major experimental challenge. In this work, we address this by using new tools and methods to dissect core protein planar polarization during *Drosophila* wing development.

## Results

### Measuring Junctional Stability of Fz-EGFP Using a New FRAP Geometry

Intercellular core protein complexes are built around an asymmetric backbone of Fz and Fmi [5, 30, 31]. In this work, as a proxy measure for core complex stability, we use fluorescence recovery after photobleaching (FRAP) to assay protein dynamics of a functional Fz-EGFP fusion created by knocking EGFP into the *fz* locus [5].

**Figure 2:**
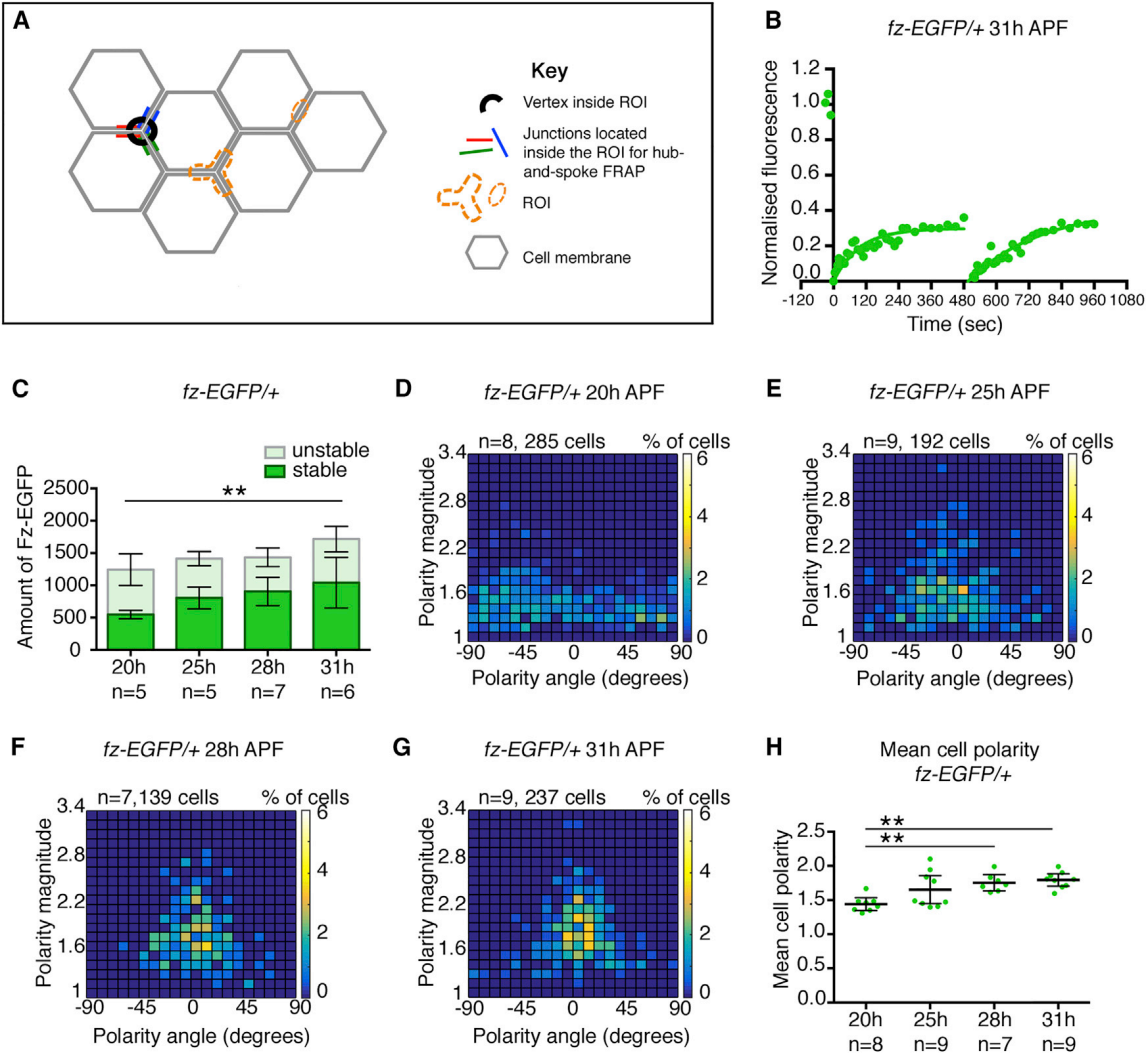
Quantitative FRAP to Measure Stable Populations of Fz-EGFP at Junctions (A) Diagram of *Drosophila* pupal wing cells showing “spot” and “hub-and-spoke” regions of interest (ROIs) used for bleaching fluorescence during FRAP. The “spot” ROI covers part of a single junction. The “hub-and-spoke” ROI covers a vertex and the junctions spreading out from that vertex. This shaped region bleaches the equivalent of the vertices and junctions of half a cell, while avoiding bleaching the cytoplasm. (B) The “hub-and-spoke” geometry does not substantially bleach overall cellular pools of Fz-EGFP. A ROI was selected and imaged 3 times prior to being bleached at 0 s; pre-bleach results are indicated as green dots prior to the 0 s time point. Recovery was allowed for 480 s (by which time Fz-EGFP recovery has reached a plateau) and then a second bleach was carried out and recovery was again allowed between 480 and 960 s. The bleached region recovered to the same extent after each bleach, consistent with the total cellular pool of Fz-EGFP not being depleted during the bleaching step and also showing that the bleaching process is not causing any cellular damage that might inhibit recovery, such as by inducing protein aggregation. Experiment was carried out at 31 hr APF. See Table S1 for y[max], half-life values, and confidence intervals. (C) Bar chart of stable and unstable amounts of Fz-EGFP at 20 hr, 25 hr, 28 hr, and 31 hr APF. n, number of wings analyzed; four regions were averaged per wing and then those results were averaged across wings. Dunnett’s multiple comparisons post hoc test was carried out on stable amounts (comparing 20 hr to other time points); 20 hr versus 31 hr ^∗∗^p = 0.0092. Other comparisons were not significant (p > 0.05). Error bars are 95% confidence intervals. See Table S2 for all statistical comparisons between the time points. (D–G) Heatmaps of angle of polarity and polarity magnitude for cells expressing Fz-EGFP in live pupal wings at (D) 20 hr, (E) 25 hr, (F) 28 hr, and (G) 31 hr APF. Data have been grouped into 20 bins on each axis; colors depict the percentage of cells in each bin. Over time, polarity magnitude increases (y axis) and the spread of polarity angles decreases (x axis). 0° represents the proximodistal axis of the wing. (H) Scatterplots showing the mean cell polarity of Fz-EGFP, calculated from the same images as the results in Figures 2D–2G. n, number of wings analyzed; the mean and 95% confidence intervals are shown. Stars indicate post hoc test results using Tukey-Kramer’s multiple comparisons test (comparing all time points to all time points); 20 hr versus 28 hr ^∗∗^p = 0.0075; 20 hr versus 31 hr ^∗∗^p = 0.0011. See also Figure S1.

In previous studies, we measured fluorescence recovery after bleaching of small regions of the cell junctions (typically an elliptical region of around 1.5 μm in length; Figures 2A and S1A) [5, 23]. This assays protein turnover in restricted locations but does not measure the dynamics of the total junctional population. We did attempt to obtain an estimate of overall proportion of stable junctional Fz by adopting a “half-cell” bleaching geometry (Figure S1B). However, this geometry could lead to substantial bleaching of the total cellular pool of Fz-EGFP, limiting the total recovery of fluorescence and thus over-estimating the stable proportion of Fz-EGFP in the junctions [32].

To better measure the stable proportion of Fz-EGFP in cell junctions, we adopted an alternative FRAP geometry that we call “hub-and-spoke” (Figure 2A). In this, the bleached region consists of a single-cell vertex (the hub) and the radiating cell junctions (the spokes). This is designed to sample protein dynamics in a population representative of all of the junctions of a cell, while avoiding bleaching of cytoplasmic protein populations.

To show that this geometry does not substantially bleach cellular pools of Fz-EGFP, we carried out a “double bleaching” experiment. A hub-and-spoke FRAP region was selected and bleached, and recovery was allowed to proceed for 8 min. The same region was then rebleached and allowed to recover. Importantly, we observed recovery to the same level following the second bleaching as after the first (Figure 2B), suggesting that this protocol gives minimal depletion of cellular pools of Fz-EGFP.

To convert the measurement of the stable *proportion* of fluorescent protein provided by FRAP into a measure of the stable *amount* of protein, we combined hub-and-spoke FRAP with quantitative measurement of total EGFP junctional fluorescence [5] (see STAR Methods). We then used this methodology to ask whether the stable amount of Fz-EGFP increases at cell junctions as core protein polarizes increases. During wing development, core protein polarization is weakest at ∼20 hr after puparium formation (APF), rising to a maximum at ∼32 hr APF [33]. We carried out measurements at 20 hr, 25 hr, 28 hr, and 31 hr APF and at the same time quantified the degree of polarization of Fz-EGFP. Between 20 and 31 hr APF, we see some increase in both the stable proportion of Fz-EGFP at junctions (Figures S1C and S1D; Table S1) and in the stable amount (Figures 2C and S1E; Table S1), in parallel with the previously reported increase in cell polarization (Figures 2D–2H).

Although significant, the increase in Fz-EGFP stability at junctions between 20 and 31 hr is surprisingly small, if sorting of core protein complexes into a polarized state is the primary driver of complex stability. According to such models (Figure 1B), as polarization proceeds, there will be an increase in stabilizing feedback interactions between complexes of the same orientation and/or a decrease in destabilizing feedback interactions between complexes of opposite orientation. The relatively weak connection between degree of cellular polarization and stability of core protein complexes suggests that core complexes have inherent stability, even when not sorted to opposite cell ends, most likely as a result of their signalosome-like organization [5].

### The Core Proteins Cooperate to Promote the Stable Junctional Localization of Fz-EGFP

Fz, Fmi, and Stbm show mutual dependency for their localization and stability at junctions [14, 15, 16, 23, 30]. However, we were previously unable to detect any role for Dsh and Pk in stabilizing core complexes at junctions [23].

**Figure 3:**
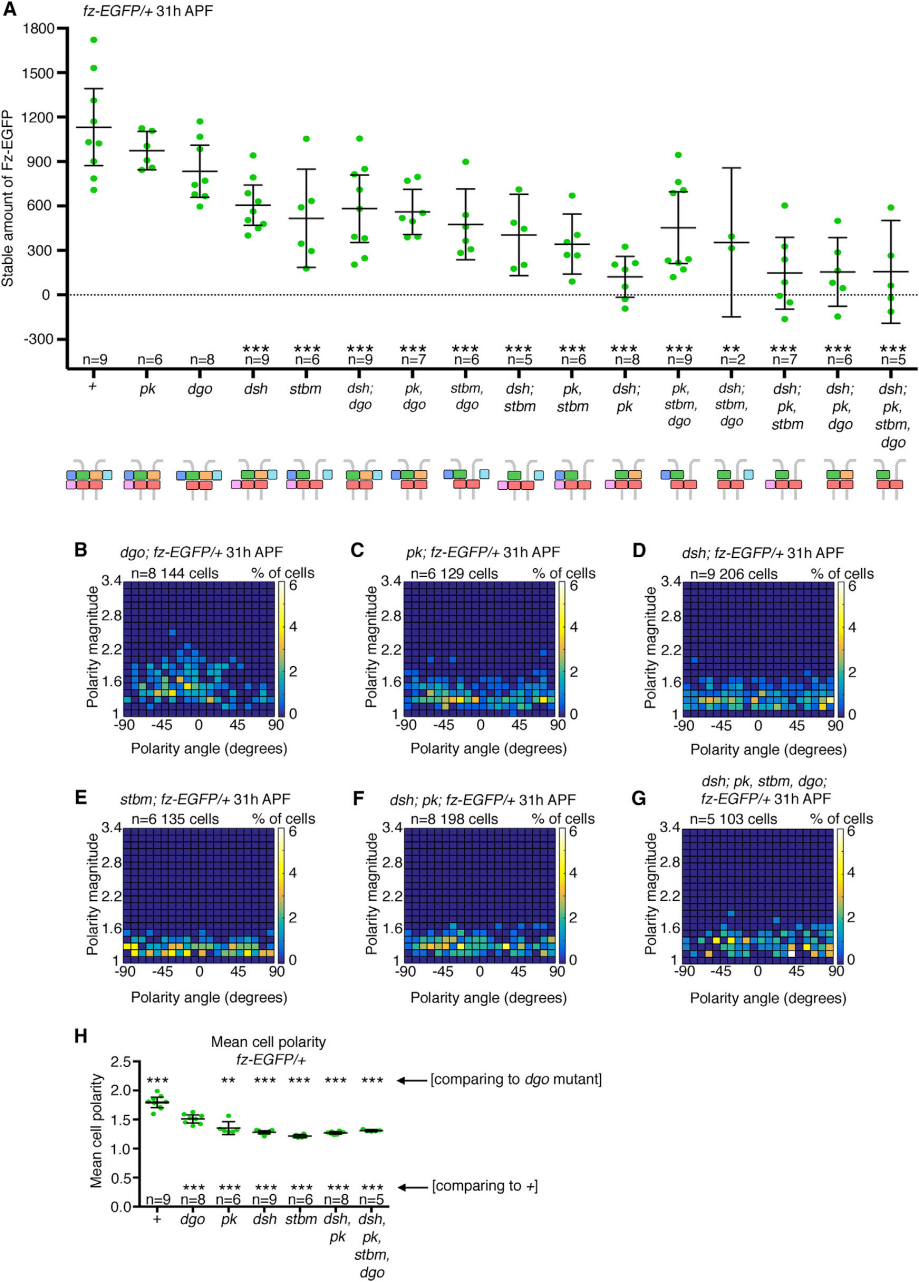
Contribution of the Core Proteins to Fz-EGFP Stability and Polarity at Cell Junctions (A) Scatterplot of the stable amounts of heterozygous Fz-EGFP at 31 hr APF in core planar polarity mutant backgrounds. See Table S1 for the full genotypes and Table S2 for the statistical comparisons between each genotype. n, number of wings analyzed; the mean and 95% confidence intervals are shown. Stars indicate significant post hoc test results comparing wild-type to mutants, using Dunnett’s multiple comparisons test; ^∗∗^p = 0.0015; ^∗∗∗^p ≤ 0.001. Note that the negative data points are due to experimental noise, generated during tracking of bleached regions. Diagrams under the graph indicate the core proteins remaining in each mutant background. Note that, in the mutant backgrounds, the actual location of the proteins in the complex may not be as depicted. (B–G) Heatmaps of angle of polarity and polarity magnitude for cells expressing Fz-EGFP in pupal wings of genotypes (B) *dgo*^*380*^, (C) *pk*^*pk-sple-13*^, (D) *dsh*^*1*^, (E) *stbm*^*6*^, (F) *dsh*^*1*^; *pk*^*pk-sple-13*^, and (G) *dsh*^*1*^; *pk*^*pk-sple-13*^, *stbm*^*6*^, *dgo*^*380*^. Data were collected from the first time point of each of the wings used in the FRAP experiments shown in (A). Data are grouped into 20 bins on each axis; colors depict the percentage of cells in each bin. (H) Scatterplots showing the mean cell polarity of Fz-EGFP, calculated from the same images as the results in (B–G). n, number of wings analyzed; the mean and 95% confidence intervals are shown. Post hoc test results using Tukey-Kramer’s multiple comparisons test (comparing all samples to each other) show that the mean cell polarity for the control is different to all of the other mutants (stars shown below scatterplots; ^∗∗∗^p ≤ 0.001). The mean cell polarity for *dgo*^*380*^ was also significantly different from all other samples (^∗∗^p = 0.0037; ^∗∗∗^p ≤ 0.001; stars shown above the scatterplots). See Table S1 for the full genotypes and Table S2 for the statistical comparisons between the genotypes. See also Figure S2.

Using quantitative hub-and-spoke FRAP, we have re-investigated the requirements for stable Fz-EGFP junctional localization. We systematically measured the total and stable amounts of Fz-EGFP at junctions at 31 hr APF in backgrounds lacking one or more core proteins (excluding Fmi, loss of which causes complete loss of Fz from junctions; Figure S2A). In these backgrounds, total levels of junctional Fz-EGFP were in a similar range (Figure S2B; Table S1). However, stable amounts of Fz-EGFP varied depending on the genotype (Figure 3A).

In the single-mutant backgrounds lacking Pk and Dgo, we did not see large changes in the stable amount of Fz-EGFP, but stable amounts were significantly reduced in the absence of Dsh and/or Stbm. A further decrease in stable amount to ∼10%–15% was seen in all multiple mutant combinations lacking both Dsh and Pk (Figures 3A and S2C–S2H; Table S1). Simultaneous loss of both Pk and Dgo also resulted in a smaller stable fraction than either mutant alone (Figure 3A; Table S1). We conclude that Dsh, Stbm, Pk, and Dgo all cooperate to promote Fz-EGFP stability at junctions and that there is functional redundancy between these proteins.

Notably, we only saw a partial correlation between the level of Fz-EGFP stability and its degree of polarization. With the exception of loss of Dgo, where weak polarization was still observed (Figures 3B and 3H), all of the other genotypes analyzed gave no detectable polarization (Figures 3C–3H); this included loss of Pk (Figures 3C and 3H), in which Fz-EGFP stability is not reduced.

Overall, our data lead to a model whereby Stbm, Dsh, Pk, and Dgo all associate with unstable Fz-Fmi-containing backbones to create stable complexes at the cell junctions with a non-stoichiometric signalosome-like organization [5], where stabilizing interactions provided by one protein can be substituted for by another protein. However, this model does not account for previous suggestions that the cytoplasmic proteins Dsh and Pk are involved in negative feedback interactions [18, 24, 26, 27].

### Using Induction of Core Protein Expression to Dissect Protein Functions

Our loss-of-function experiments reveal the net long-term effect of the absence of a protein on Fz-EGFP stability. However, interpretation of such final phenotypes is difficult in the absence of temporal knowledge regarding how protein behaviors change upon removal or addition of a pathway component.

**Figure 4:**
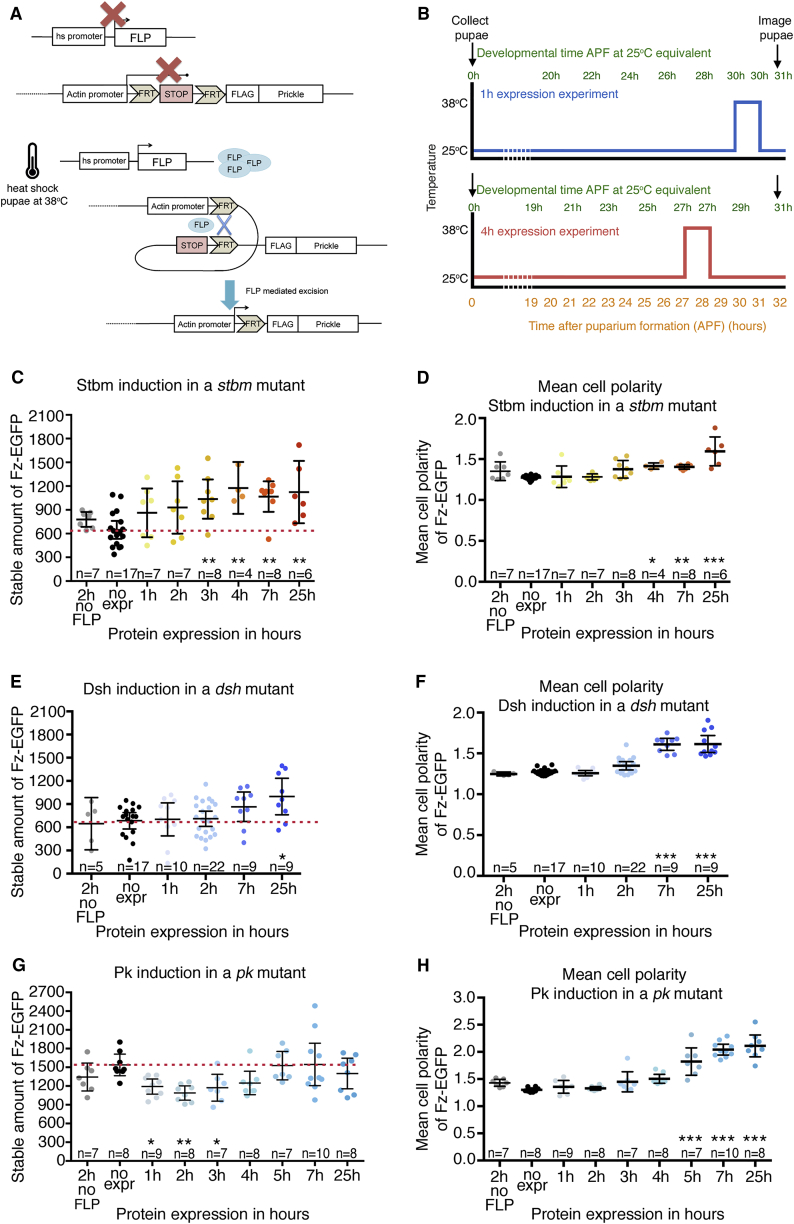
Induction of Expression Experiments Reveal Net Stabilizing Functions for Stbm and Dsh but a Destabilizing Function for Pk (A) Diagram illustrating induction of expression of core proteins, showing FLAG-Pk as an example. (B) Timeline showing heat shock induction of protein expression relative to developmental time, with examples of 1 hr and 4 hr induction experiments. Note, based on timing of trichome initiation, we observe that developmental time is halted during heat shock at 38°C. (C, E, and G) Stable amount of Fz-EGFP after induction of expression of (C) Stbm, (E) Dsh, or (G) Pk. FRAP was performed at 31 hr APF, with heat shock induction of protein expression between 1 hr and 25 hr prior to imaging. The no FLP control (gray circles in each graph) did not carry *hsFLP* but was heat shocked 2 hr prior to imaging, whereas the no expression control carried *hsFLP* but was not heat shocked. The mean and 95% confidence intervals are shown. Stars on the graphs indicate significant results using Dunnett’s multiple comparisons test, comparing the no expression stable amount to that of the other conditions. For Pk induction in (G), no expression versus 1 hr ^∗^p = 0.0335, no expression versus 2 hr ^∗∗^p = 0.003, and no expression versus 3 hr ^∗^p = 0.0369. Student’s t test was used to compare no FLP 2 hr expression to 2 hr expression: ^∗^p = 0.0236. See Table S1 for full genotypes and Table S2 for all comparisons. (D, F, and H) Scatterplots showing the mean cell polarity of Fz-EGFP in live pupal wings, after induction of (D) Stbm, (F) Dsh, and (H) Pk. Data were collected from the first FRAP images; n, number of wings analyzed; the mean and 95% confidence intervals are shown. Stars on the graphs indicate significant results using Dunnett’s multiple comparisons test, comparing the mean vector polarity of the no expression control to that of the other conditions; ^∗^p ≤ 0.05; ^∗∗^p ≤ 0.01; and ^∗∗∗^p ≤ 0.001. Note that, after 24 hr induction, Stbm and Dsh rescue polarity to the same level as seen in *fz-EGFP/+* at 31 hr APF (compare to Figure 2H; measured polarity is not significantly different between these genotypes). After 24 hr of Pk expression, the measured polarity is slightly stronger than seen in *fz-EGFP/+*, although the reasons for this are not known. See Table S2 for all comparisons. See also Figure S3.

To investigate the functions of the core proteins in a more refined way, we designed experiments that involve induction of expression of a core protein in the physiological range, followed by assaying the effect on Fz-EGFP stability and polarization. We used previously characterized transgenes that express core proteins under control of an *Actin5C* promoter, with the promoter separated from the core protein coding sequence by a cassette containing a polyadenylation site that can be excised upon heat shock when in the presence of *hsFLP* (Figure 4A) [34, 35]. Our previous work indicated that heat shocks of 1 to 2 hr at 38°C are sufficient to activate expression throughout the wing and that expression under the *Actin5C* promoter is able to provide physiological activity [35]. To confirm this, we carried out immunoblotting and quantified the levels of Stbm, Dsh, and Pk produced: normal expression levels are provided by transgenes within 1 to 2 hr post-induction for Dsh and 2 to 3 hr for Pk, whereas Stbm is visible after 2 hr but does not achieve normal expression levels until 7 hr after induction (Figures S3A–S3F).

For consistency, Fz-EGFP stable amounts and level of polarization were always assayed at 31 hr APF (or the equivalent age for pupae raised at temperatures other than 25°C). Pupae mutant for the gene of interest, but carrying the appropriate inducible transgene, were then heat shocked at different time points prior to 31 hr APF to give different length periods of induced protein expression (Figure 4B). In this way, it is possible to follow the time course of activity of a core protein in promoting Fz-EGFP stability and polarization. As controls, we also treated the same genotypes to a heat shock but in the absence of *hsFLP*. This will not activate expression from the *Actin5C* transgene and should reveal any effects of the heat shock alone.

### Induction of Expression Reveals Net Stabilizing Functions for Stbm and Dsh but a Destabilizing Function for Pk

We first investigated the effects of induction of Stbm and Dsh expression. Absence of both leads to a decrease in Fz-EGFP stability (Figure 3A). Induction of either protein in its corresponding mutant background rescues this reduced stability, with increasing periods of expression post-induction leading to increased Fz-EGFP stability (Figures 4C and 4E) and polarization (Figures 4D and 4F). In neither background did we see a significant effect of heat shock alone on Fz-EGFP stability (Figures 4C and 4E).

Despite these similarities in the effects of Stbm and Dsh induction, there were also differences. Fz-EGFP stability increased within 3 hr of Stbm induction (Figure 4C), with significant polarization being observed by 4 hr (Figure 4D). Conversely, Dsh induction only resulted in a significant increase in Fz-EGFP stability by 25 hr (Figure 4E), despite significant polarization being seen by 7 hr (Figure 4F). We consider likely explanations in the Discussion.

We then carried out induction of Pk and Dgo, loss of either of which shows a negligible effect on Fz-EGFP stability (Figure 3A). Notably, induction of Dgo in a *dgo* background did not lead to consistent changes in Fz-EGFP stability, suggesting that Dgo does not play a major role in complex stability in the wing (Figures S3G and S3H).

In contrast, induction of Pk resulted in a decrease in Fz-EGFP stability, peaking around 2 hr after protein induction, followed by a return to the pre-induction level of stability (Figures 4G and S3I). This recovery of Fz-EGFP stability is accompanied by increasing Fz-EGFP polarization (Figure 4H).

We conclude that, during the process of core protein polarization, the primary functions of Dsh and Stbm are to stabilize Fz-containing complexes at junctions. Conversely, induction of Pk reveals a net destabilizing function.

### Induced Pk Requires Dsh Activity to Destabilize and Stbm Activity to Stabilize Fz

Whereas our loss-of-function analysis suggested only stabilizing functions for Pk (Figure 3A), our induction experiments revealed a destabilizing activity (Figure 4G). To reconcile these observations, we combined induction of Pk expression with epistasis experiments, in which another core protein was absent. In particular, we analyzed the effect of inducing Pk expression in the absence of the stabilizing factors Stbm or Dsh. We reasoned that, if Pk acted by promoting or inhibiting the stabilizing function of Stbm or Dsh, then this effect would be blocked in their absence.

**Figure 5:**
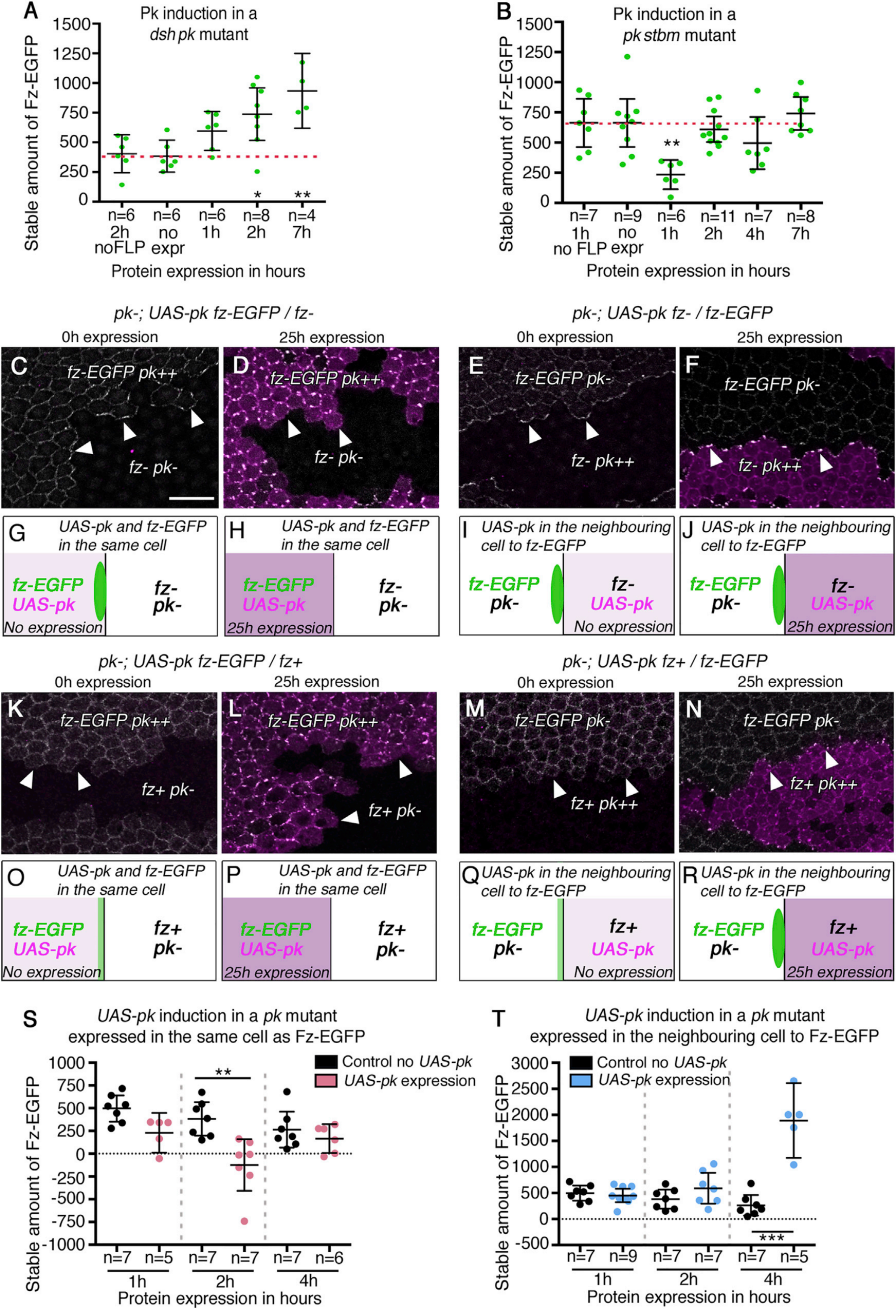
Induced Pk Requires Dsh Activity to Destabilize Fz-EGFP and Stbm Activity to Stabilize Fz-EGFP (A and B) Stable amount of Fz-EGFP after induction of expression of Pk in (A) a *dsh*^*1*^; *pk*^*pk-sple-13*^ background and (B) a *pk*^*pk-sple-13*^*stbm*^*6*^ background. FRAP was performed at 31 hr APF. The no FLP control did not carry *hsFLP* but was heat shocked prior to imaging, whereas the no expression control carried *hsFLP* but was not heat shocked. The mean and 95% confidence intervals are shown. A post hoc Tukey-Kramer’s multiple comparisons test was used to compare all the conditions. (A) Results of: no FLP 2 hr versus 2 hr ^∗^p = 0.0274; 0 hr (no expression) versus 2 hr ^∗^p = 0.0175; no FLP 2 hr versus 7 hr ^∗∗^p = 0.0022; and 0 hr (no expression) versus 7 hr ^∗∗^p = 0.0014. (B) Results of: no FLP 1 hr versus 1 hr ^∗∗^p = 0.0045 and 0 hr (no expression) versus 1 hr ^∗∗^p = 0.0024. Note that 1 hr versus 2 hr ^∗∗^p = 0.007 and 1 hr versus 7 hr ^∗∗∗^p = 0.0003 were also statistically significant comparisons. See Table S1 for full genotypes and Table S2 for all comparisons. (C–J) Images and cartoon summaries of pupal wings with twin clones of cells expressing Fz-EGFP and elevated Pk next to *fz*^*P21*^ mutant cells (C, D, G, and H) or *fz*^*P21*^ mutant cells expressing elevated Pk next to Fz-EGFP-expressing cells (E, F, I, and J). Wings were in a *pk*^*pk-sple-13*^ mutant background, and expression of *UAS-pk* was controlled using *GAL4/GAL80*^*ts*^. (C, E, G, and I) Pupae were raised at 18°C for 60 hr, giving no *UAS-pk* expression. (D, F, H, and J) Pupae were raised at 29°C for 25 hr, activating *UAS-pk* expression. Immunolabeled images show GFP (in white) and Pk (in magenta). Arrowheads indicate Fz-EGFP on clone boundaries. Note Pk overexpression in Fz-EGFP-expressing tissue results in clustering of Fz-EGFP at cell boundaries away from the clone boundary (D). The scale bar represents 10 μm. (K–R) Images and cartoon summaries of pupal wings with twin clones of cells expressing Fz-EGFP and elevated Pk next to Fz-expressing cells (K, L, O, and P) or cells expressing Fz and elevated Pk next to Fz-EGFP-expressing cells (M, N, Q, and R). Wings were in a *pk*^*pk-sple13*^ mutant background, and expression of *UAS-pk* was controlled using *GAL4/GAL80*^*ts*^. (K, M, O, and Q) Pupae were raised at 18°C for 60 hr, giving no *UAS-pk* expression. (L, N, P, and R) Pupae were raised at 29°C for 25 hr, activating *UAS-pk* expression. Immunolabeled images show GFP (in white) and Pk (in magenta). Arrowheads indicate Fz-EGFP on clone boundaries. Note Pk overexpression in Fz-EGFP-expressing tissue results in clustering of Fz-EGFP at cell boundaries away from the clone boundary (L). (O–R) Cartoon summary showing the locations of Fz on clone boundaries in the mosaic experiments in (K)–(N). (S and T) The stable amount of Fz-EGFP after short-term expression of Pk in the same cells (S) or neighboring cells (T). FRAP was performed at 28 hr APF. Pk expression was induced by shifting pupae from 18°C to 29°C for 1 hr, 2 hr, or 4 hr prior to FRAP. n, number of wings analyzed; the mean and 95% confidence intervals are shown. Stars on the graphs indicate significant results using the Šídák’s multiple comparisons test. The control samples are the same in both experiments. (S) 2 hr control versus 2 hr of Pk expression in the same cell as Fz ^∗∗^p = 0.0024. The negative mean value for Fz-EGFP stability after 2 hr of induction is most likely due to experimental noise generated during tracking of bleached regions. (T) 4 hr control versus 4 hr of Pk expression in the neighboring cell to Fz ^∗∗∗^p < 0.001. See Table S1 for full genotypes and Table S2 for all comparisons. See also Figure S4.

Notably, when Pk was induced in pupae lacking Dsh activity, instead of seeing a destabilization of Fz-EGFP, we instead saw stabilization above the baseline level within 2 hr (Figure 5A). Nevertheless, in the absence of Dsh, as expected, cells were unable to polarize (Figures S4A, S4B, and S4E). This ability of Pk to stabilize Fz-EGFP in the absence of Dsh is consistent with our loss-of-function analysis (Figure 3A), where we observed only low Fz-EGFP stability in the absence of both Dsh and Pk but significantly higher stability in the absence of only Dsh.

Conversely, when Pk is induced in the absence of Stbm activity, we see a destabilization of Fz-EGFP that is both more rapid and larger than that seen upon induction when Stbm is present (Figure 5B; compare Figure 4G). This destabilization is only temporary and is no longer observed at 2 hr after induction, consistent with our earlier observation that, at steady-state, Fz-EGFP stability is similar in both a *stbm* background and a *pk stbm* background (Figure 3A). Note that again Fz-EGFP does not polarize (Figures S4C–S4E), consistent with Stbm activity being essential for polarization.

These results indicate that the ability of Pk to destabilize Fz-EGFP is dependent on the presence of Dsh, but not of Stbm. As Dsh binds to and colocalizes with Fz in core protein asymmetric complexes (Figure 1A), we speculated that the destabilizing function of Pk might require Pk directly acting on Fz-Dsh complexes in the same cell. However, when Dsh is not present, Pk induction results in an increase in Fz stability. We suggest that, in this case, Pk may be acting by interacting with its binding partner Stbm to stabilize Fz-EGFP in neighboring cells (Figure 1A).

We therefore carried out mosaic experiments, in which we could assess whether Pk was acting in the same cell as Fz-Dsh or in neighboring cells. We first generated genetically mosaic tissue, in which cells expressing Fz-EGFP were juxtaposed to cells lacking Fz expression, in a background lacking Pk expression. In this situation, Fz-EGFP localizes to the boundary between Fz-expressing and non-expressing cells (Figures 5C, 5E, 5G, and 5I). We then induced prolonged high-level Pk expression in either the Fz-EGFP-expressing cells or the Fz non-expressing cells, using the *GAL4/GAL80*^*ts*^ temperature-sensitive expression system [36]. Consistent with our model, when Pk was expressed in the Fz-EGFP-expressing cells, Fz-EGFP levels at the clone border were reduced (Figures 5D and 5H). Moreover, when Pk was expressed in the adjacent cells, Fz-EGFP levels on the apposing cell junctions were maintained and possibly increased (Figures 5F and 5J), consistent with Pk interactions with Stbm on one side of the boundary leading to stabilization of Fz-EGFP on the other side.

It has previously been suggested that Pk destabilizes Stbm in response to the presence of Fz in the same membrane [24]; however, our experiments expressing Pk in cells lacking Fz activity (Figures 5F and 5J) are unable to test this. We therefore repeated the same experiment but in this case juxtaposing cells expressing Fz-EGFP to cells expressing untagged endogenous Fz. In the absence of Pk expression, we once more saw Fz-EGFP on the boundary between expressing and non-expressing cells (Figures 5K, 5M, 5O, and 5Q), albeit more weakly than when Fz is lacking in the adjacent cells. Overexpression of Pk in the Fz-EGFP-expressing cells again resulted in a loss of Fz-EGFP from the clone boundary (Figures 5L and 5P), whereas expression in the adjacent Fz-expressing cells resulted in a striking punctate accumulation of Fz-EGFP on the clone boundary (Figures 5N and 5R). Therefore, we did not detect a destabilizing activity of Pk acting via Stbm.

We wanted to confirm that the decreases or increases in Fz-EGFP levels that we observed were not an artifact caused by long-term overexpression of Pk. In particular, Pk overexpression leads to clustering of core proteins at junctions [26], and in cells expressing Fz-EGFP and Pk, this might be leading to indirect sequestration of Fz-EGFP from the clone boundary (e.g., as may occur in Figures 5D and 5L). We therefore also carried out experiments in which expression was only induced for a short period of time and carried out FRAP to measure the effect on Fz-EGFP stability before any gross relocalization was observed. Significantly, short-term expression of Pk in cells expressing Fz-EGFP initially caused a loss of stable Fz-EGFP by 2 hr, followed by a return of stability by 4 hr (Figure 5S). Conversely, expression in neighboring cells resulted in a strong stabilization of Fz-EGFP by 4 hr (Figure 5T).

Our epistasis and mosaic experiments therefore allow us to separate two functions of Pk, providing evidence that Pk can destabilize Fz-EGFP when present in the same cell, in a Dsh-dependent manner, and can also stabilize Fz-EGFP by acting in a Stbm-dependent manner in neighboring cells.

### Destabilization of Fz-EGFP by Pk Requires Dynamin and Rab5 Activity

We were interested to understand the molecular mechanism of the Dsh-dependent destabilization of Fz-EGFP by Pk. It has been suggested that competitive binding of Pk to Dsh might result in displacement either of Dsh from Fz [26], or of Dsh from Dgo [27], with a consequent loss of Fz stability. However, we think these mechanisms unlikely, as mass action kinetics dictate that competitive binding interactions will only proceed rapidly if either the factor being displaced is only weakly bound or the displacing factor is present at much higher concentrations. We know from our previous studies that Dsh and Dgo are stably associated with Fz at junctions [5], which is not consistent with weak binding interactions. Moreover, Dgo does not show high Fz stabilizing activity in the wing (Figure 3A), casting doubt on whether it could be a key feedback component. Furthermore, the levels of Pk expressed under control of the *Actin5C* promoter when maximal Fz-EGFP destabilization is seen (2 hr post-induction) are similar to wild-type levels (Figures S3E and S3F), indicating that Pk is not present in great excess over Dsh or Dgo.

Alternatives to a competitive binding mechanism are allosteric effects, where, for instance, Pk binding to Dsh might alter its conformation and hence its properties, and/or a catalytic mechanism whereby the presence of Pk alters local enzyme activity, leading to destabilization of Fz. A possible catalytic destabilizing mechanism would be triggering Fz endocytosis.

**Figure 6:**
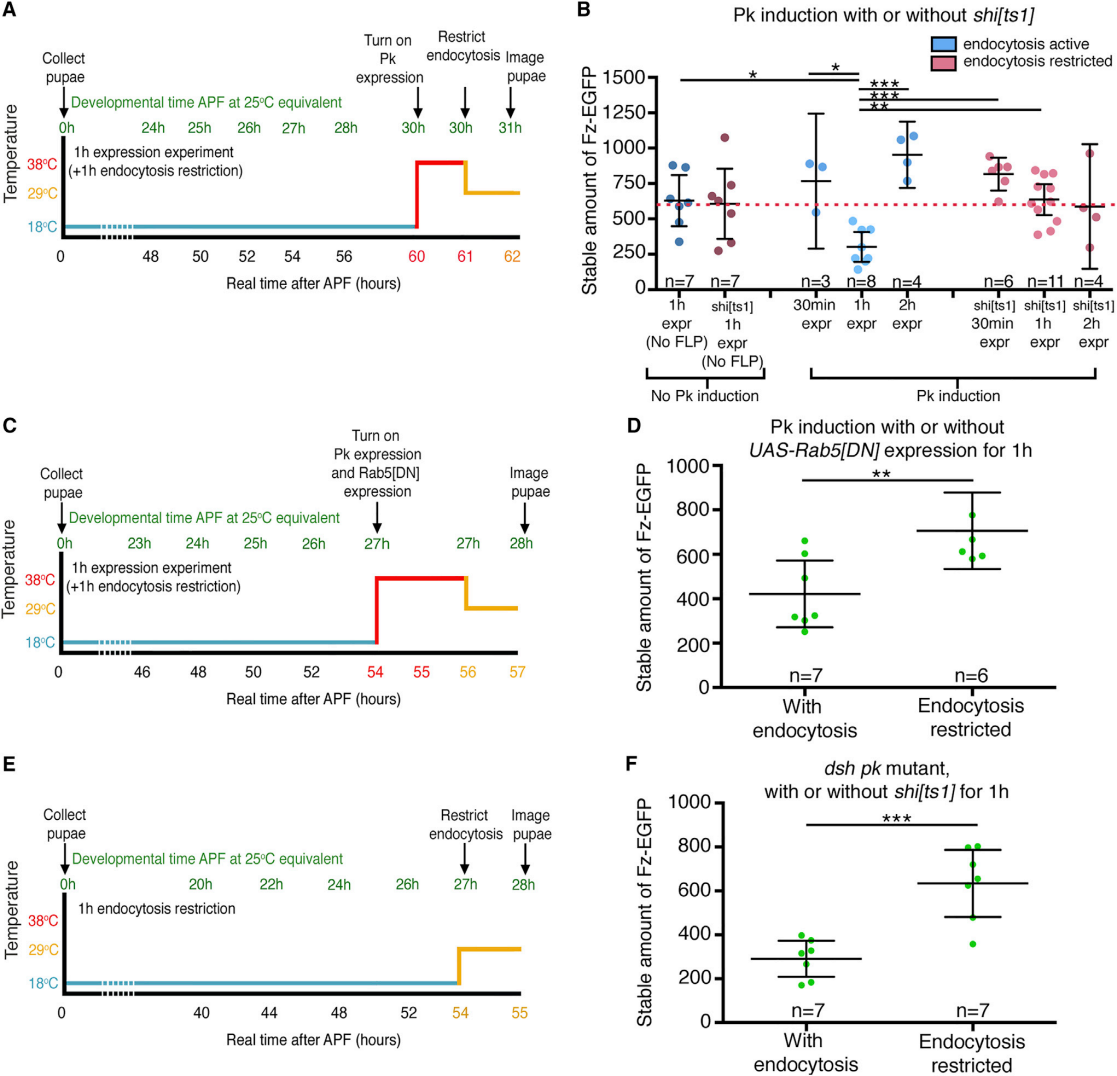
Destabilization of Fz-EGFP by Pk Requires Active Endocytosis (A) Timeline of a Pk induction experiment conducted when endocytosis was restricted, using a *shi* temperature-sensitive background (*shi*^*ts1*^). Pupae are initially aged at 18°C to allow active endocytosis to occur (blue line). Pk expression is turned on using a heat shock (38°C for 1 hr, red line). Endocytosis is then restricted by shifting the pupae to 29°C (yellow line). Equivalent developmental time in hours at 25°C is in green as a comparison to the real time in hours shown below the graph. (B) Induction of Pk in *pk*^*pk-sple-13*^ background, with either active (blue points) or restricted (red points) endocytosis using the temperature-sensitive allele of *shi*^*ts1*^. Scatterplots show the stable amount of Fz-EGFP at junctions. The mean and 95% confidence intervals are shown. In no FLP controls, pupae are heat shocked but Pk expression is not induced. Stars on the graphs indicate significant post hoc test results of the ANOVA using Tukey-Kramer’s multiple comparisons test, comparing all conditions to the rest; ^∗^p ≤ 0.05; ^∗∗^p ≤ 0.01; and ^∗∗∗^p ≤ 0.001. See Table S1 for full genotypes and Table S2 for all comparisons. (C) Timeline of a Pk induction experiment conducted when endocytosis was active or restricted, using *Act≫Gal4* to express dominant-negative *UAS*-*Rab5*^*S43N*^. Pupae are initially aged at 18°C in the presence of endocytosis (blue line). Pk expression and *UAS*-*Rab5*^*S43N*^ are turned on using a heat shock (38°C for 2 hr, red line). Pupae are then aged at 29°C for 1 hr before imaging (yellow line). Equivalent developmental time in hours at 25°C is in green as a comparison to the real time in hours shown below the graph. (D) Induction of Pk in *pk*^*pk-sple-13*^ background, with either active or restricted endocytosis for 1 hr using the dominant-negative UAS-*Rab5*^*S43N*^. Scatterplots show the stable amount of Fz-EGFP at junctions; n, number of wings analyzed; the mean and 95% confidence intervals are shown. Stars on the graph indicate the result of a t test comparing the stable amounts of Fz-EGFP; ^∗∗^p = 0.0096. See Table S1 for full genotypes. (E) Timeline of a *dsh*^*1*^*pk*^*pk-sple-13*^ double-mutant experiment conducted when endocytosis was active or acutely restricted, using a *shi* temperature-sensitive background (*shi*^*ts1*^). Pupae are initially aged at 18°C to allow active endocytosis to occur (blue line). Endocytosis is then restricted by shifting the pupae to 29°C (yellow line). Equivalent developmental time in hours at 25°C is in green as a comparison to the real time in hours shown below the graph. (F) A *dsh*^*1*^*pk*^*pk-sple-13*^ double-mutant experiment conducted when endocytosis was active or acutely restricted for 1 hr, using a *shi* temperature-sensitive background (*shi*^*ts1*^). Scatterplots show the stable amount of Fz-EGFP at junctions; n, number of wings analyzed; the mean and 95% confidence intervals are shown. Stars on the graph indicate the result of a t test comparing the stable amounts of Fz-EGFP; ^∗∗∗^p = 0.0004. See Table S1 for full genotypes. See also Figure S5.

To test for a role of endocytosis in Fz destabilization upon Pk induction, we carried out experiments in a background carrying a temperature-sensitive allele of *Drosophila* dynamin (*shi*^*ts1*^), which is widely used as a tool to acutely block endocytosis [37, 38]. Prior to Pk induction via heat shock, the pupae were raised at the permissive temperature (18°C) and endocytosis was active; then, after the heat-shock, the pupae were transferred to 29°C, which is a restrictive temperature that will reduce dynamin activity and endocytosis (Figure 6A).

When carried out in a background with wild-type dynamin activity, this regime results in a large and rapid destabilization of Fz-EGFP upon Pk induction (Figure 6B). This effect is not seen either if *hsFLP* is absent, thus preventing Pk induction, or in a *shi*^*ts1*^ background, where dynamin activity and endocytosis was blocked (Figure 6B). As a further control, we confirmed that Pk is normally produced and delivered to junctions in the absence of dynamin activity (Figures S5A and S5B).

As another test of whether Fz-EGFP destabilization might be dependent on endocytosis, we induced expression of a dominant-negative form of the endocytic protein Rab5 at the same time as inducing Pk. This also interfered with the ability of Pk to destabilize Fz-EGFP at junctions (Figures 6C and 6D). These results support the hypothesis that Pk induces removal of Fz-EGFP via endocytosis.

Interestingly, under conditions where Pk induction leads to very large reductions in the stable amount of Fz-EGFP at junctions (e.g., Figures 5B and 6B), there was no accompanying large reduction in the total amount of Fz-EGFP at junctions (Table S1), suggesting that destabilized Fz-EGFP is rapidly recycled to re-engage in complex formation. Consistent with this, by immunolabeling, we were unable to detect any increase in cytoplasmic puncta of Fz-EGFP, EGFP-Dsh, or Fmi, following induction of Pk expression (Figures S5C–S5H, S5K, and S5L). We also did not observe gross recruitment of the endocytic proteins clathrin light chain or Rab5 to the cell junctions following Pk induction (Figures S5I–S5L), implying that Pk is not triggering large-scale non-specific endocytosis.

We speculated that Dsh might normally protect Fz from constitutive endocytosis and that induced Pk blocks this protective activity. If this were true, we reasoned that the low stability of Fz-EGFP observed in the absence of Dsh and Pk (Figure 3A) would be due at least partly to endocytosis. We therefore measured the stable fraction of Fz-EGFP in a *dsh*; *pk* background, either in the absence or presence of *shi*^*ts1*^ at the restrictive temperature (Figure 6E). Notably, reducing endocytosis partly restored Fz-EGFP stability (Figure 6F). Therefore, we propose that Dsh normally protects Fz from endocytosis and induction of Pk blocks this activity.

## Discussion

**Figure 7:**
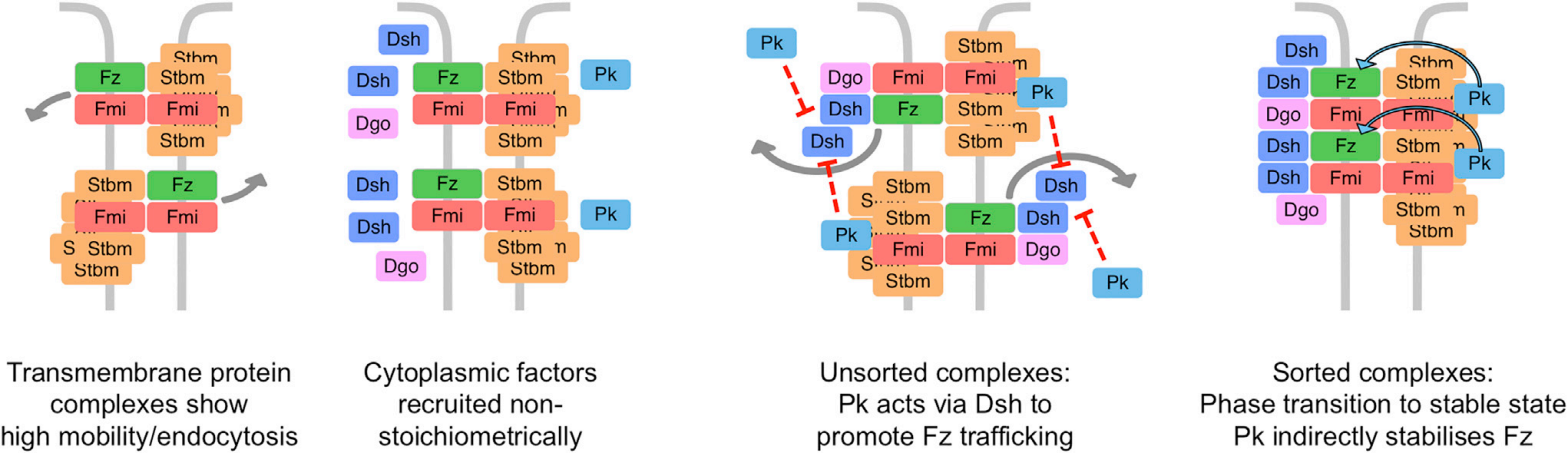
Model for Role of Pk in Feedback Amplification of Planar Polarity In the absence of cytoplasmic factors, Fz-containing complexes at cell junctions show high mobility (left panel), at least in part due to active endocytosis (arrows). Cytoplasmic factors are non-stoichiometrically recruited by the transmembrane core proteins (left-center panel). These proteins participate in feedback amplification interactions (right-center panel) with Pk destabilizing Fz in the same cell via a Dsh-dependent mechanism that results in Fz endocytosis (gray arrows). At the same time, the cytoplasmic factors stabilize complexes, most likely via a phase transition driven by multivalent binding interactions between the core proteins (right panel), with Pk acting via Stbm to stabilize Fz in neighboring cells (blue arrows).

In this study, we investigate core planar polarity pathway function by combining quantitative measurements of protein dynamics with both loss-of-function genetics and induction of gene function. Taken together, our results support the following model for planar polarization (Figure 7): (1) polarized cell-cell communication is mediated by asymmetric core protein complexes built around an asymmetric Fz-Fmi:Fmi backbone; (2) Stbm, Dsh, Pk, and Dgo promote complex stability at junctions (with partial redundancy); and (3) in addition to its stabilizing function (mediated via Stbm in neighboring cells), Pk can also destabilize Fz-containing complexes in a Dsh-dependent manner in the same cell.

Previously, relying on analysis of single mutant genotypes, we were unable to detect direct roles for Dsh and Pk in either stabilizing or destabilizing Fz-containing complexes [23]. It is now apparent that these functions were masked in part due to each protein having multiple functions. Our findings illustrate the power of temporal manipulation of gene activity to unmask essential functions [39].

Based on our recent discovery that stable fractions of Stbm, Dsh, Pk, and Dgo can associate with Fz and Fmi non-stoichiometrically [5], we propose that core protein complexes have a signalosome-like organization whereby weak multivalent protein-protein interactions between the complex components promote a phase transition into a stable state [6]. Such an arrangement would provide complexes with inherent stability at cell junctions. Our present findings show that, in the absence of stabilization by the other complex components, Fz is highly mobile at junctions, in part due to being actively endocytosed.

Once core proteins are stably localized at cell junctions, feedback interactions can then sort them into polarized domains. We do not know whether there is only one feedback interaction or multiple interactions; however, experimental and theoretical studies looking at single cell polarization suggest that combinations of multiple feedbacks increase robustness (e.g., [40, 41]).

One such feedback interaction appears to be mediated via the ability of Pk to destabilize Fz in the same cell. Dynamin and Rab5 are required to see this destabilization of Fz, supporting an endocytic mechanism. This may involve a direct interaction between Pk and Dsh, given that they are known to bind in vitro [26, 27]. We propose two possible (non-exclusive) mechanisms: (1) Pk interacts with Dsh to block a protective function of Dsh on Fz (most likely multimerization into stable complexes), leading to Fz endocytosis via a constitutive mechanism. This is consistent with our observation of constitutive Fz endocytosis in the absence of Dsh and Pk activity, and (2) binding of Pk to Dsh-Fz complexes results in a post-translational modification of Dsh or Fz by an enzyme that is itself recruited by Pk. Possible modifications include phosphorylation, which might induce a further destabilizing conformational change or recruitment of further enzymes or endocytic adaptors, or the change could be ubiquitination of Fz or Dsh, promoting internalization [42, 43, 44].

During normal development, we envisage that it is the population of Pk bound to Stbm that mediates destabilizing feedback interactions between core complexes and leads to their sorting. However, our experiments show that “free” Pk can also destabilize Fz in the absence of Stbm, and we speculate that this may be important for maintaining plasticity by ensuring there is always some turnover of complexes.

It was recently found that, in addition to binding Pk and localizing it to proximal cell edges [16, 45], Stbm also promotes Pk degradation by the proteasome [24, 29]. We speculate that this might serve to prevent a large buildup of Pk, which would continuously destabilize Fz. However, interestingly, we find that induction of Pk expression in the absence of Stbm only induces a transient destabilization of Fz (Figure 5B). In this case, the cells appear to show adaptation, whereby they initially respond to a signal but then adapt and become unresponsive [46]. If free Pk is causing Fz to be constitutively endocytosed, it is possible that a homeostatic mechanism acts to progressively damp down this response. Alternatively, it is possible that Pk can also stabilize complexes, even in the absence of Stbm, for instance, via interactions with Fmi [47], but this stabilization acts on a longer timescale than its ability to destabilize Fz.

Another interesting observation is that, whereas induction of both Dsh and Stbm expression leads to stabilization of Fz-EGFP, Stbm induction leads to rapid stabilization (Figures 4C and 4D), whereas Dsh induction shows much slower stabilization (Figures 4E and 4F). This occurs despite induction of Stbm showing a slower time course of protein production than Dsh (Figures S3A–S3D). We speculate that this may be because of the different interactions Stbm and Dsh have with Pk. When Stbm is induced, we predict that Stbm associates with intercellular complexes on the opposite side of junctions to Fz and both recruits Pk and promotes Pk degradation by the proteasome [24, 29]. This results in stabilization of Fz at junctions both intercellularly by Stbm-Pk but also reduces free Pk, which could destabilize Fz intracellularly. The balance of interactions is therefore toward Fz stabilization, which rises rapidly (Figure 4C). When Dsh is induced, we predict it will associate with Fz and stabilize it at junctions. However, Pk opposes this stabilization by acting to destabilize Fz and thus causing high mobility of both Fz and Dsh. There is thus competition between the stabilizing activity of Dsh and the destabilizing activity of Pk. The competition only ceases once Dsh and Pk are largely sequestered to opposite cell ends, at which point Fz stability rises (Figure 4E).

Notwithstanding our evidence for an essential role for Pk in mediating feedback interactions that amplify planar polarity, it is known that polarity (as revealed by effects on trichome formation) can propagate from repolarizing boundaries through *pk* mutant tissue [35, 48]. Nevertheless, in the absence of Pk, the core proteins cannot detectably planar polarize. We infer that, in the absence of Pk, there is at least a small difference in core protein distribution or activity that can be passed from cell to cell and which is sufficient to activate the trichome polarization machinery.

In conclusion, our data support Pk-mediating negative interactions that destabilize Fz during feedback amplification of planar polarity in the *Drosophila* wing (Figure 7). Stbm, Dsh, Pk, and Dgo also play roles in stabilizing core complexes, most likely by promoting multivalent protein-protein interactions. Together, these processes are essential for polarized subcellular distribution of core planar polarity protein complexes.

## STAR★Methods

### Key Resources Table

**Table:** 

REAGENT or RESOURCE	SOURCE	IDENTIFIER
**Antibodies**		

Rabbit anti-GFP, affinity purified	Abcam	cat#ab6556; RRID: AB_305564
Mouse monoclonal anti-actin AC-40	Sigma-Aldrich	cat#A4700; RRID: AB_476730
Mouse monoclonal anti-Fmi #74	Developmental Studies Hybridoma Bank [14]	RRID: AB_2619583
Rat anti-Pk, affinity purified	David Strutt [49]	N/A
Rabbit anti-Dsh, affinity purified	David Strutt [49]	N/A
Rabbit anti-Stbm	Tanya Wolff [50]	N/A

**Chemicals, Peptides, and Recombinant Proteins**		

16% paraformaldehyde solution (methanol free)	Agar Scientific	cat#R1026
Triton X-100	VWR	cat#28817.295; CAS: 9002-93-1
Glycerol	VWR	cat#284546F; CAS: 56-81-5
DABCO	Fluka	cat#33480; CAS: 280-57-9
Normal Goat Serum	Jackson Labs	cat#005-000-121
Halocarbon 700 Oil	Halocarbon Products	CAS: 9002-83-9

**Experimental Models: Organisms/Strains**		

*fmi*^*E59*^	Tadashi Uemura [14]	FlyBase: FBal0101421
*stbm*^*6*^	Tanya Wolff [51]	FlyBase: FBal0062423
*dsh*^*V26*^	Norbert Perrimon [52]	FlyBase: FBal0003140
*dsh*^*1*^	Bloomington Drosophila Stock Center	FlyBase: FBal0003138; RRID: BDSC_5298
*pk*^*pk-sple13*^	David Gubb [53]	FlyBase: FBal0060943
*dgo*^*380*^	Suzanne Eaton [25]	FlyBase: FBal0141190
*shi*^*ts1*^	Bloomington Drosophila Stock Center	FlyBase: FBal0015610; RRID: BDSC_7068
*fz-EGFP*	David Strutt [5]	N/A
*P[acman]-EGFP-dsh*	David Strutt [5]	N/A
*pActP*>*STOP*>*dsh-ECFP*	David Strutt [35]	N/A
*pActP*>*STOP*>*FLAG-stbm*	This paper	N/A
*pActP*>*STOP*>*FLAG-pk*	This paper	N/A
*pActP*>*STOP*>*FLAG-dgo*	This paper	N/A
*UAS-Pk attP{VK00031}*	This paper	N/A
*tub-CFP-Rab5*	Suzanne Eaton [54]	N/A
*sqh-Clc::mCherry*	Thomas Lecuit [55]	N/A
*pUAS-Rab5*^*S43N*^	Bloomington Drosophila Stock Center	FlyBase: FBti0150344; RRID: BDSC_42703
*hs-FLP*	Bloomington Drosophila Stock Center	FlyBase: FBti0002044; RRID: BDSC_6
*FLP22*	Bloomington Drosophila Stock Center	FlyBase: FBti0000785; RRID: BDSC_8862
*Ubx-FLP*	Bloomington Drosophila Stock Center	FlyBase: FBti0150334; RRID: BDSC_42718
*Actin-GAL4*	Bloomington Drosophila Stock Center	FlyBase: FBti0012293; RRID: BDSC_4414
*tubulin-Gal80*^*ts*^	Bloomington Drosophila Stock Center	FlyBase: FBti0027796; RRID: BDSC_7019
*Actin*>*y+*>*GAL4*	Bloomington Drosophila Stock Center	FlyBase: FBti0012290; RRID: BDSC_3953
*Ubi-mRFP-nls*	Bloomington Drosophila Stock Center	FlyBase: FBti0129786; RRID: BDSC_30852

**Software and Algorithms**		

NIS Elements AR version 4.60	Nikon	N/A
Image Lab version 4.1	BioRad Laboratories	N/A
ImageJ version 2.0.0	https://fiji.sc	N/A
MATLAB_R2014b	Mathworks	N/A
GraphPad Prism version 7.0c	GraphPad Software	N/A
G^∗^Power version 3.1	http://www.gpower.hhu.de	N/A
Packing Analyzer	Suzanne Eaton [33]	N/A
Polarity measurement scripts (MATLAB)	David Strutt [5]	N/A

### Contact for Reagent and Resource Sharing

Further information and requests for resources and reagents should be directed to and will be fulfilled by the Lead Contact, David Strutt (d.strutt@sheffield.ac.uk).

### Experimental Model and Subject Details

*Drosophila melanogaster* flies were grown on standard cornmeal/agar/molasses media at 18°C or 25°C, unless otherwise described.

Fly strains are described in the Key Resources Table. *fmi*^*E59*^, *fz*^*P21*^, *stbm*^*6*^, *pk*^*pk-sple13*^, *dsh*^*V26*^ and *dgo*^*380*^ are null alleles. *dsh*^*1*^ gives a strong planar polarity phenotype, but functions normally in *wingless* signaling. *shi*^*ts1*^ is a temperature sensitive allele, which is functional at the permissive temperature (18°C) and is less active at the restrictive temperature (29°C).

*fz-EGFP* is a knockin of EGFP into the endogenous *fz* locus and *P[acman]-EGFP-dsh* is a genomic rescue construct. Both are expressed at similar levels to the endogenous genes, and fully rescue mutant phenotypes [5]. *pActP*>*STOP*>*FLAG-stbm*, *pActP*>*STOP*>*FLAG-pk* and *pActP*>*STOP*>*FLAG-dgo* were generated by using PCR to introduce FLAG-tags at the N-termini of the coding sequences and inserting the final products into the vector *pActP*>*STOP*>*PolyA*. *UAS-pk* was generated by ΦC31-mediated recombination into the *VK31* landing site. Other transgenes were *pActP*>*STOP*>*dsh-ECFP* [35], *tub-CFP-Rab5* [54] and *sqh-Clc::mCherry* [55]. *pUAS-Rab5*^*S43N*^ was obtained from Bloomington Stock Centre (*w; P{w[+mC]*=*UAS-Rab5.S43N}2*) (see also Key Resources Table).

Transgenics were generated by Genetivision and BestGene.

### Method Details

#### Fly genetics

To express *dsh-ECFP*, *FLAG-stbm*, *FLAG-pk* and *FLAG-dgo*, *hsFLP* or *FLP22* were used, and pupae were heat shocked for 1 hr at 38°C at the indicated times to excise the *FRT-STOP-FRT* cassette. On the basis of when trichomes subsequently emerge, we observe that development is halted for the period of the heat shock. The start time of protein expression was varied to ensure FRAP was always conducted at the same developmental time at 31 hr APF. The genotype for the heat shock control did not carry *FLP* but was heat shocked under the same conditions, 2 hr prior to imaging. *UAS-Rab5*^*S43N*^ was expressed after excision of the *FRT-STOP-FRT* cassette from *Actin*>*y+*>*GAL4* using *hsFLP* following a 2 hr heat shock at 38°C.

Mitotic clones were induced using the FLP/FRT system and *Ubx-FLP*. For twin clone experiments with *fz-EGFP* and *UAS-pk*, mitotic clones were induced with *Ubx-FLP*, and *pk* expression was controlled using *Actin-GAL4, tub-GAL80*^*ts*^. Flies were raised at 18°C and shifted to 29°C to allow expression of *UAS-pk* for the indicated times. For FRAP on clone boundaries, tissue lacking *fz-EGFP* was marked using *Ubi-mRFP-nls*.

#### Immunostaining and antibodies

Unless otherwise indicated, pupal wings were dissected at 28 hr APF at 25°C. Pupae were placed in a drop of 4% paraformaldehyde in PBS and the pupal cuticle was removed. Pupae were fixed for 30-45 min at room temperature, prior to dissection of the pupal wing from the pupal carcass. Wings were transferred into PBS containing 0.2% Triton X-100 (PTX) and 10% normal goat serum to block prior to antibody incubation. Wings were incubated with antibodies overnight at 4°C, in PTX with normal goat serum, and washes were in PTX. After immunostaining wings were post-fixed in 2% paraformaldehyde in PTX for 30 min and mounted in 10% glycerol, 1xPBS, containing 2.5% DABCO (pH7.5). Primary antibodies for immunostaining were affinity purified rabbit anti-GFP (ab6556, Abcam, UK), affinity-purified rat anti-Pk [49] and mouse monoclonal anti-Fmi (Flamingo #74, DSHB [14]).

#### Western blotting

For pupal wing westerns, 28-30 hr APF pupal wings were dissected directly into sample buffer. Two pupal wing equivalents were loaded per lane. Westerns were probed with affinity-purified rat anti-Pk [49], affinity purified rabbit anti-Dsh [49], rabbit anti-Stbm [50] and monoclonal mouse anti-actin (AC-40, Sigma, UK). A BioRad ChemiDoc XRS+ was used for imaging. Images were quantified using the Gel Analysis plug-in in ImageJ.

#### Imaging

Fixed pupal wings were imaged on a Nikon A1R GaAsP confocal microscope using a 60x NA1.4 apochromatic lens, with a pixel size of 70 nm, and the pinhole was set to 1.2 AU. 9 Z-slices separated by 150 nm were imaged, and then the 3 brightest slices around junctions were selected and averaged for each channel in ImageJ.

For live imaging, white prepupae were collected and aged for 31 hr at 25°C (or the equivalent time at different temperatures). Briefly, a small piece of cuticle was removed from above the pupal wing, and the exposed wing was mounted in a drop of Halocarbon 700 oil in a glass-bottomed dish. Images were taken below vein 5, as this is the flattest region in our preparations. For FRAP analysis, images were 256 × 256 pixels, with a pixel size of 100 nm, and a pinhole of 1.2 AU. “Hub-and-spoke” ROIs of approximately 4 μm^2^ were selected, that covered a vertex and 3 half-cell edges. Three pre-bleach images were taken at 2 frames/sec, and ROIs were then bleached using a 488 nm Argon laser at 80% with 8 passes (1 s total time), which resulted in 50%–60% bleaching. Immediately following bleaching, 5 images were taken at 5 s intervals, followed by 10 images at 10 s intervals, 10 images at 15 s intervals and 8 images at 30 s intervals. Laser power was adjusted to maintain constant power between different imaging sessions.

If only EGFP was being imaged, a 488nm laser and a long pass GFP filter was used. For imaging both EGFP and mRFP, a 488nm laser and a 525-550 band pass filter was used to detect GFP and a 561nm laser and a 550-595 band pass filter to detect mRFP. For the *UAS-pk* and *shi*^*ts1*^ experiments, pupae were imaged on a heated stage at 29°C.

### Quantification and Statistical Analysis

#### FRAP processing

For data analysis, ImageJ was used to manually reselect up to 4 bleach regions in each image for each time point. The laser off background was subtracted, and the values were then corrected for acquisition bleaching and normalized against the average of the prebleach values. Data were then plotted on an xy graph using Prism (v7 Graphpad), bleached regions within the same wing were averaged and a one-phase exponential curve was fitted for each wing. Multiple wings were then combined and an exponential association curve was fitted. An extra-sum-of-squares F test was used to compare curve plateaux (y[max]).

To determine the stable amount of Fz-EGFP in the ROIs, the mean intensity of the ROIs from the three pre-bleach images was measured in ImageJ, and averaged per wing. The intensity was then corrected for distance from the coverslip as previously described [5], and this value was then multiplied by the stable fraction (1-y[max]) for each wing. The stable amounts were then averaged across wings, and results were plotted on a scatter graph along with the mean and 95% confidence intervals.

#### Statistics

The overall intensities, and stable and unstable amounts for multiple genotypes were compared using a one-way ANOVA, to take into account the sample variation across the genotypes analyzed and to avoid multiple t test analysis. Post hoc tests were used to compare individual samples: Dunnett’s multiple comparison test was used to compare the control to the rest of the genotypes in the experiment; Tukey-Kramer’s multiple comparison test to compare all genotypes within an experiment; and Šídák’s multiple comparison test was sometimes used to compare genotypes pairwise. Where a post hoc test was used this is described in the Figure legends, and multiplicity adjusted p values calculated in Prism are reported on the graph as asterisks (p < 0.5^∗^, p < 0.01^∗∗^, p < 0.001^∗∗∗^), and in Tables S1 and S2.

Based on the mean intensity and standard deviation of a control set of wings, we aimed for a sample size of at least 6 wings per genotype. This would allow detection of differences of 20% in the means, in a pairwise comparison, with a power of 0.8 and α 0.05 (using G^∗^Power). In practice the power was lower than this, as standard deviations were larger for some genotypes, multiple genotypes were compared in each experiment, and in many cases the desired number of wings was not achieved, due to the constraints of timed pupa collections of complex genotypes.

Each experiment was performed on multiple wings from different pupae, which represent biological replicates (n, number of wings). For each wing, 4 ROIs were selected for FRAP analysis, and these were treated as technical replicates and were averaged per wing to produce a y[max] and a stable amount per wing. Data was excluded if the ROI recovery curve failed the ‘replicates test for lack of fit’ in GraphPad Prism, or if the wing moved out of focus during the course of imaging. In total 14 wings were discarded across all the genotypes.

#### Polarity measurement

Polarity measurements were taken from the pre-bleach images that were used for FRAP. Wings were aligned along the proximodistal wing axis based on wing vein orientation, and membrane masks were generated using Packing Analyzer [33]. Polarity magnitude (maximum asymmetry ratio on a cell-by-cell basis) and polarity angle were determined using previously described MATLAB scripts [5]. To visualize the polarity magnitude compared to the polarity angle, data from multiple wings were combined, and heatmaps were created using MATLAB. Data was grouped into 20 bins on each axis, with each bin representing a unique polarity angle and magnitude. Colors depict the percentage of cells in each bin, and 0° represents the proximodistal axis of the wing.

## Author Contributions

S.J.W. and H.S. performed experiments, S.J.W. and K.H.F. performed data analysis, and D.S. and H.S. designed the experiments and wrote the paper.

## Supplemental Information

Document S1. Figures S1–S5

Table S1. Quantitative FRAP Data, Related to Figures 2, 3, 4, 5, and 6 Genotypes for each FRAP experiment, along with the timepoints at which the pupal wings were imaged are shown, along with the number (n) of wings imaged for each genotype, the total fluorescence intensity, the stable amounts, the y[max] (plateau), the half-life and the 95% confidence intervals for each of the data sets. The figure panel relating to the results is also indicated.

Table S2. Detailed Statistical Comparisons, Related to Figures 2, 3, 4, 5, and 6 Detailed results for the statistical tests carried out on the data shown in the figures are shown. The statistical test (either ANOVA or t-test is indicated), as well as the names of the post hoc tests. These include the Dunnett's multiple comparisons test (for comparing the control to the other experimental conditions), Tukey-Kramer's multiple comparisons test (for comparing all conditions to all others), and the Šídák's multiple comparisons test (to compare pre-selected pairs of conditions within an experiment). The table also shows the mean difference and 95% confidence intervals. Stars indicate statistical significance ^∗^P≤0.05 ^∗∗^P≤0.01 and ^∗∗∗^P≤0.001. The figure panel relating to each statistical test is also indicated.

Document S2. Article plus Supplemental Information
