# Supplementary material for: A Dual Function for Prickle in Regulating Frizzled Stability during Feedback-Dependent Amplification of Planar Polarity
Source: Curr Biol. 2017 Sep 25;27(18):2784–2797.e3. doi: 10.1016/j.cub.2017.08.016 (PMC5628951; doi:10.1016/j.cub.2017.08.016)
Supplement: Document S1. Figures S1–S5 [file mmc1.pdf]

Current Biology, Volume *27*

Supplemental Information

A Dual Function for Prickle in Regulating Frizzled Stability during Feedback-Dependent Amplification of Planar Polarity

Samantha J. Warrington, Helen Strutt, Katherine H. Fisher, and David Strutt

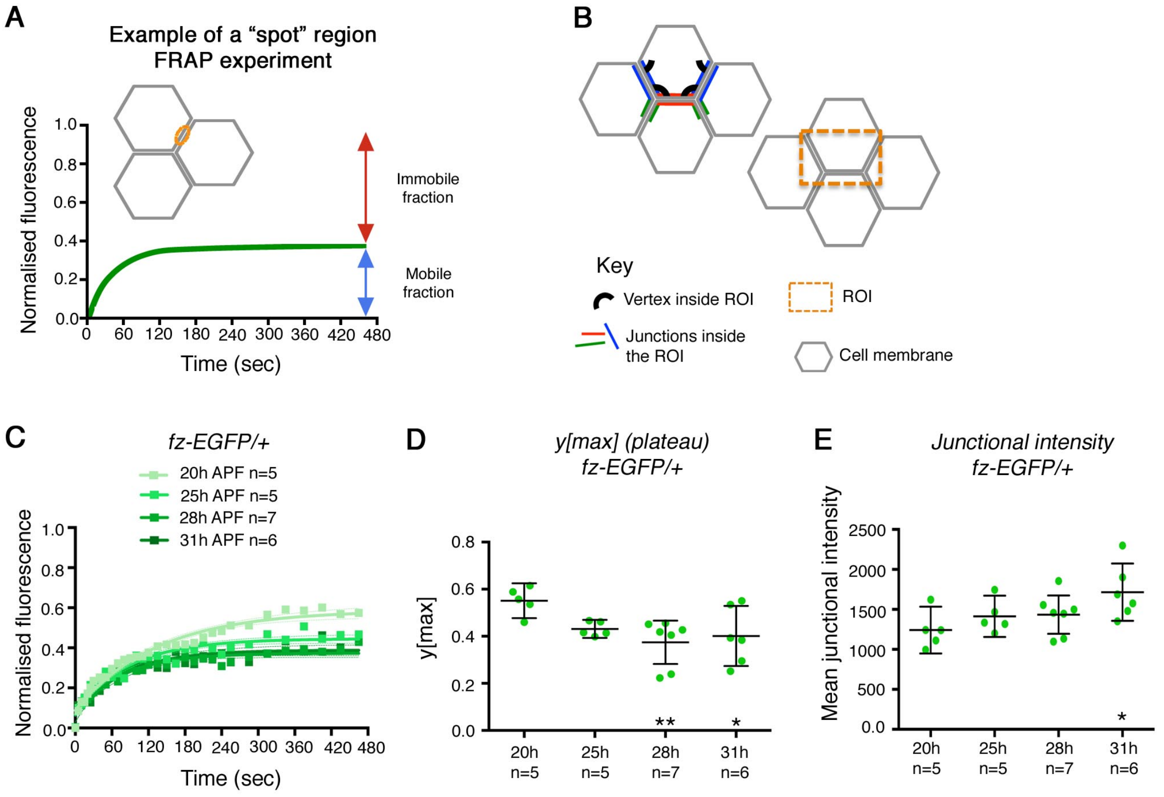

Figure S1. Quantitative FRAP of Fz-EGFP. Related to Figure 2

(A) Diagram of an idealised spot-bleach experiment. After bleaching of the region of interest (ROI, orange ellipse) at the start of the experiment, fluorescence gradually recovers in the bleached region as a result of exchange of any mobile bleached molecules within the ROI for unbleached material from outside the ROI, by either diffusion or intracellular trafficking. The degree of recovery achieved reveals the size of the mobile fraction of material (blue arrow) whereas the proportion of fluorescence that does not recover reveals the size of the immobile fraction (red arrow). The rate of recovery of fluorescence is revealed by the slope of the recovery curve and provides a measure of the rate of protein turnover within the ROI.

(B) Diagram showing "half-cell" ROI used for bleaching fluorescence during FRAP. This shaped region bleaches the equivalent of the vertices and junctions of at least an entire cell, while also bleaching significant regions of cytoplasm, possibly resulting in depletion of the total cellular pool of fluorescent protein from which fluorescence recovery can occur.

(C) Hub-and-spoke FRAP recovery curves of heterozygous Fz-EGFP at 20h, 25h, 28h and 31h APF, fitted to a single exponential equation. 95% confidence intervals for the curve are also plotted as dotted lines in the same colour. Plateaux comparison of all of the curves: extra sum-of-squares F test result $P \leq 0.001$. Rate comparison of all the curves: extra sum-of-squares F test result $P=0.0279$.

(D) Scatter plots showing the FRAP y[max] (plateau) of Fz-EGFP recovery in live pupal wings at 20h, 25h, 28h and 31h APF. Analysed from the same images as the results in Figure 2C. n = number of wings with 4 regions averaged per wing. Green dots are the averaged results from each wing; the mean and 95% confidence intervals are shown. Post hoc test results using Dunnett's multiple comparisons test (comparing 20h APF column to all columns) 20h vs. 28h **P= 0.0085, 20h vs. 31h *P= 0.0318.

(E) Scatter plots showing the mean junctional intensity of Fz-EGFP in live pupal wings at 20h, 25h, 28h and 31h APF. Analysed from the same images as the results in Figure 2C. n = number of wings with 4 regions averaged per wing. Green dots are the averaged results from each wing; the mean and 95% confidence intervals are shown. Post hoc test results using Dunnett's multiple comparisons test (comparing 20 h column to all columns) *P=0.0241.

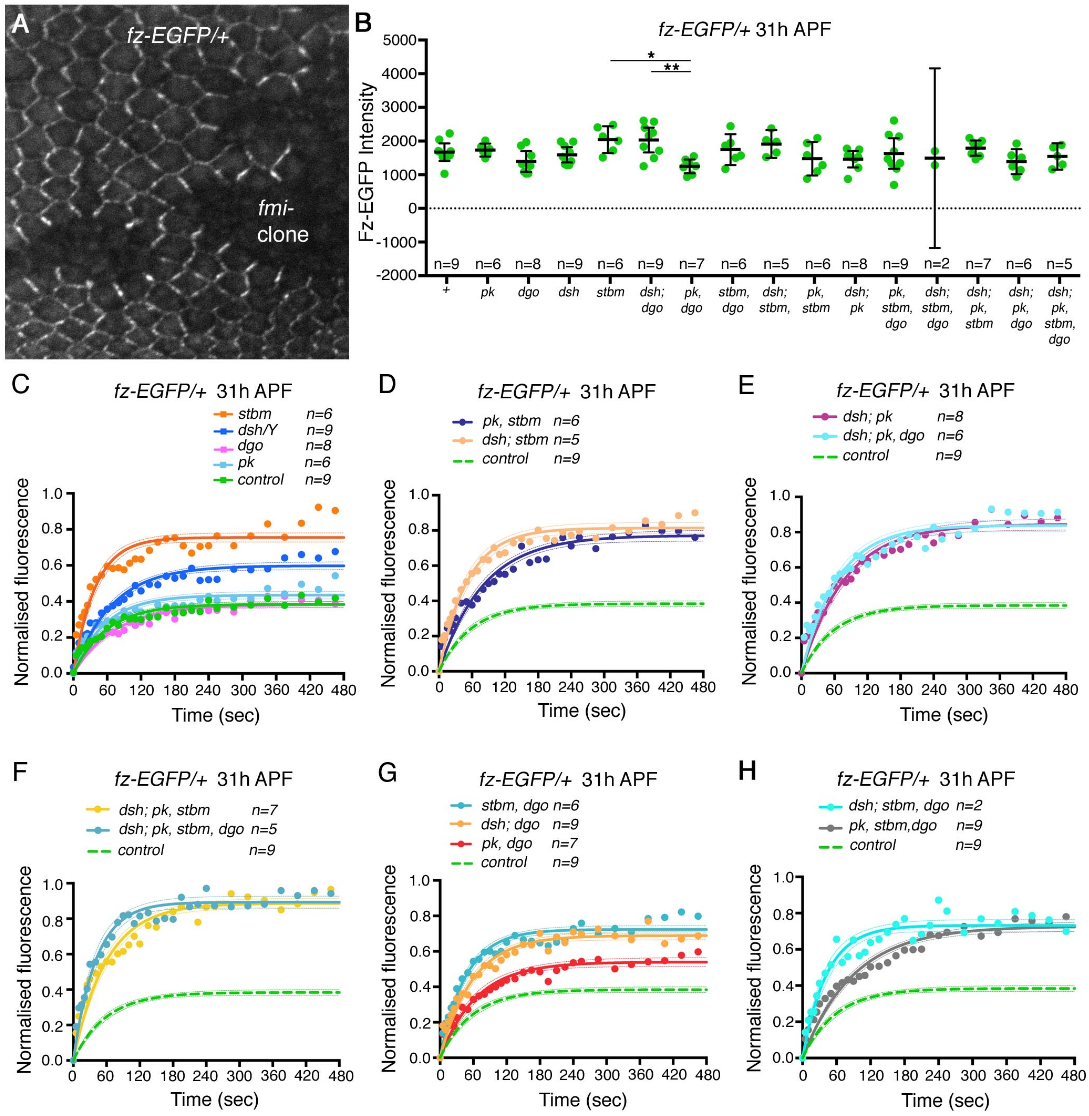

Figure S2. Amounts and fluorescence recovery after photobleaching of Fz-EGFP at junctions in different core pathway genotypes. Related to Figure 3

(A) Image of Fz-EGFP fluorescence in 31h APF pupal wing carrying *fmi^{E59}* mutant clones. No Fz-EGFP is visible at junctions in *fmi* mutant tissue.

(B) Scatter plot showing Fz-EGFP intensity in the control (+) and core planar polarity mutant backgrounds. Error bars are 95% confidence intervals. A post hoc Tukey-Kramer's multiple comparisons test (comparing all genotypes to each other) was conducted; significant comparisons were *$stbm^{6}$* vs. *$pk^{pk\text{-}sple\text{-}13}$ dgo^{380}* (*P=0.020) and *dsh^{1} dgo^{380}* vs. *$pk^{pk\text{-}sple\text{-}13}$ dgo^{380}* (**P=0.007). See Table S1 for the full genotypes and Table S2 for the statistical comparisons between the genotypes.

(C-H) FRAP recovery curves of Fz-EGFP at 31h APF, in core planar polarity mutant backgrounds. Each curve is fitted to a single exponential equation. 95% confidence intervals for the curves are indicated as dotted lines in the same colour. *fz-EGFP/+* control is shown as a solid green line in (C) and as a dotted green line in (D-H). The mutant genotypes in each graph are: (C) *$stbm^{6}$* (orange); *dsh^{1}* (dark blue); *dgo^{380}* (pink); *$pk^{pk\text{-}sple\text{-}13}$* (light blue). (D) *$pk^{pk\text{-}sple\text{-}13}$ $stbm^{6}$* (navy); *dsh^{1} $stbm^{6}$* (peach). (E) *dsh^{1} $pk^{pk\text{-}sple\text{-}13}$* (purple); *$dsh^{1}$ $pk^{pk\text{-}sple\text{-}13}$ dgo^{380}* (light blue). (F) *dsh^{1} $pk^{pk\text{-}sple\text{-}13}$ $stbm^{6}$* (yellow); *dsh^{1} $pk^{pk\text{-}sple\text{-}13}$ $stbm^{6}$ dgo^{380}* (blue). (G) *$stbm^{6}$ dgo^{380}* (blue); *dsh^{1} dgo^{380}* (orange); *$pk^{pk\text{-}sple\text{-}13}$ dgo^{380}* (red). (H) *dsh^{1} $stbm^{6}$ dgo^{380}* (turquoise); *$pk^{pk\text{-}sple\text{-}13}$ $stbm^{6}$ dgo^{380}* (grey). See Table S1 for the full genotypes.

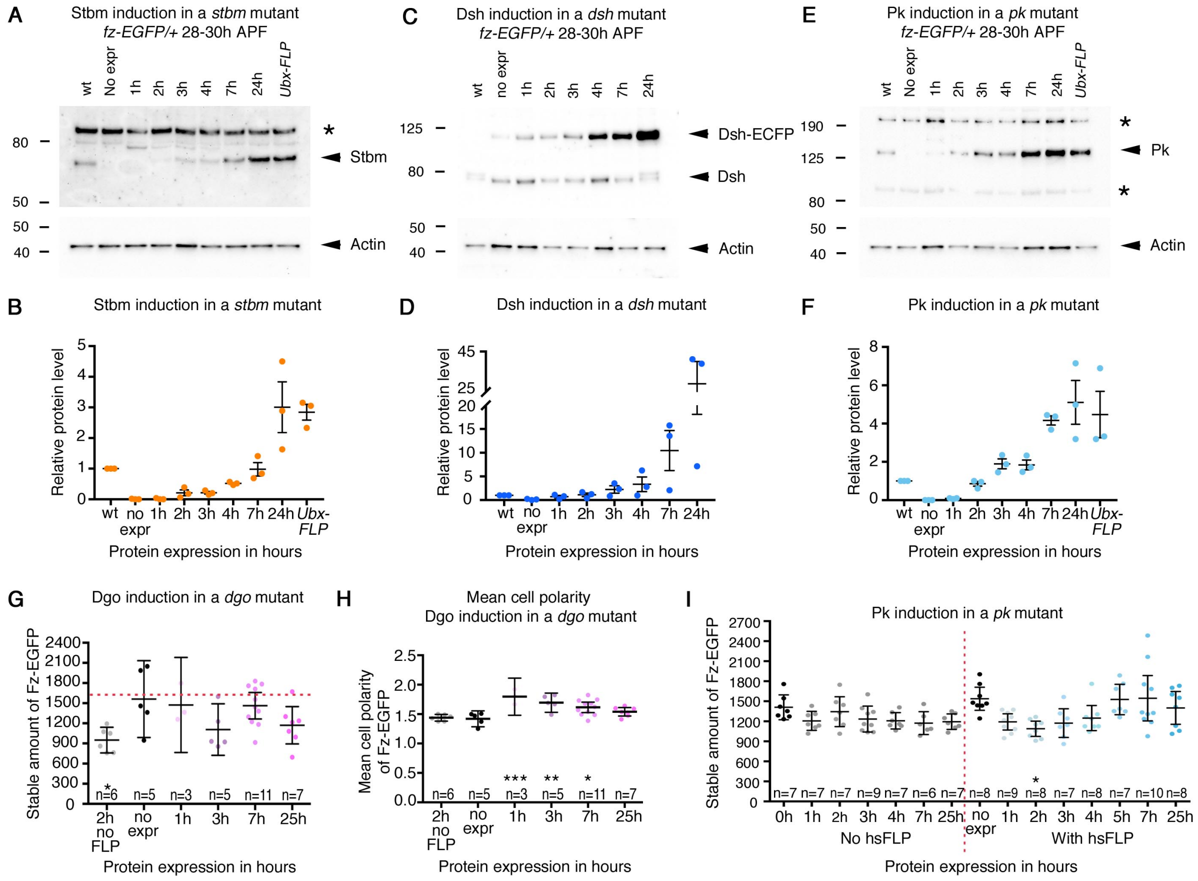

Figure S3. Protein induction profiles for Stbm, Dsh and Pk, and effects of Dgo and Pk induction on Fz-EGFP stability. Related to Figure 4

(A,C,E) Immunoblots of Stbm (A), Dsh (C) or Pk (E) protein levels and Actin levels after indicated time of induction of expression, in 28-30 h APF pupal wing extracts, compared to a wild-type (*w^{1118}*) control. (A) *hs-FLP; Actin>>FLAG-stbm, $stbm^6$/ $stbm^6$; fz-EGFP/+* flies or *Ubx-FLP; Actin>>FLAG-stbm, $stbm^6$/ $stbm^6$; fz-EGFP/+* flies for long-term expression. (C) *dsh^1, FLP22; Actin>>Dsh-ECFP; fz-EGFP/+* flies. (E) *hs-FLP; Actin>>FLAG-pk, $pk^{pk\text{-}sple\text{-}13}$/ $pk^{pk\text{-}sple\text{-}13}$; fz-EGFP/+* flies or *Ubx-FLP; Actin>>FLAG-pk, $pk^{pk\text{-}sple\text{-}13}$/ $pk^{pk\text{-}sple\text{-}13}$; fz-EGFP/+* flies for long-term expression. Arrows indicate Stbm, Dsh or Pk protein, and the asterisks indicate non-specific bands on the immunoblots. (A,B) Stbm is detectable 2h after induction, and reaches wild-type levels after 7h. By 24h the protein is overexpressed, and levels are at a steady state, comparable to those seen with Ubx-FLP. (C,D) Note that Dsh-ECFP is larger than endogenous Dsh, and leaky expression is detectable in the absence of heat shock (13% of endogenous levels). Wild-type levels are achieved 1-2h after induction using heat shock, and protein levels continue to increase over the time course. The *dsh^1* allele encodes a protein of the correct size that is not phosphorylated (see absence of upper band). Interestingly when Dsh-ECFP is over-expressed after 24 h of expression, the phosphorylation of the protein encoded by *dsh^1* is restored, possibly due to sequestration by Dsh-ECFP. (E,F) Pk reaches wild type levels 2-3h after induction, and is overexpressed by 7h, when steady state levels are achieved.

(B,D,F) Quantitation of Stbm, Dsh and Pk protein levels relative to Actin levels, for the immunoblots shown in A, C and E. Quantitations were from 3 biological replicates, and graphs show mean and sem.

(G) Stable amount of Fz-EGFP after induction of expression of Dgo. FRAP was performed at 31h APF, with heat shock induction of protein expression between 1h and 25h prior to imaging. The no FLP controls (grey circles in each graph) did not carry *hs-FLP* but were heat shocked 2 h prior to imaging, while the no expression controls carried *hs-FLP* but were not heat shocked. The mean and 95% confidence intervals are shown. Stars on the graphs indicate significant results using Dunnett's multiple comparisons test, comparing the no-expression stable amount to that of the other conditions, 2h expression, no FLP vs. no expression *P= 0 .0116. See Table S1 for full genotypes, Table S2 for all comparisons.

(H) Scatter plots showing the mean cell polarity of Fz-EGFP in live pupal wings, after induction of Dgo. Data were collected from the first FRAP images. n = number of wings analysed, mean and 95% confidence intervals are shown. Stars on the graphs indicate significant results using Dunnett's multiple comparisons test, comparing the mean cell polarity of the no expression control to that of the other conditions: 0h vs. 1h ***P=0.0003, 0h vs. 3h **P=0.002 and 0h vs. 7h *P=0.011. Note that the no expression and the no FLP controls retain a low degree

of polarity even in the absence of *dgo*^{380}, similar to the *dgo*^{380} mutant (Figure 3B,H). See Table S1 for full genotypes, Table S2 for all comparisons.

(I) Stable amount of Fz-EGFP after induction of expression of Pk. Samples are the same as for Figure 4G, but additional controls are shown. FRAP was performed at 31h APF, with heat shock induction of protein expression between 1h and 25h prior to imaging. The no FLP controls (grey circles) did not carry *hs-FLP* but were heat shocked at various times prior to imaging, while the no expression control carried *hs-FLP* but was not heat shocked. The mean and 95% confidence intervals are shown. Student's t-tests were used to compare no FLP controls to expression (with FLP) at each timepoint; no FLP 2h expression to 2h expression: *P=0.0236. See Table S1 for full genotypes, Table S2 for all comparisons.

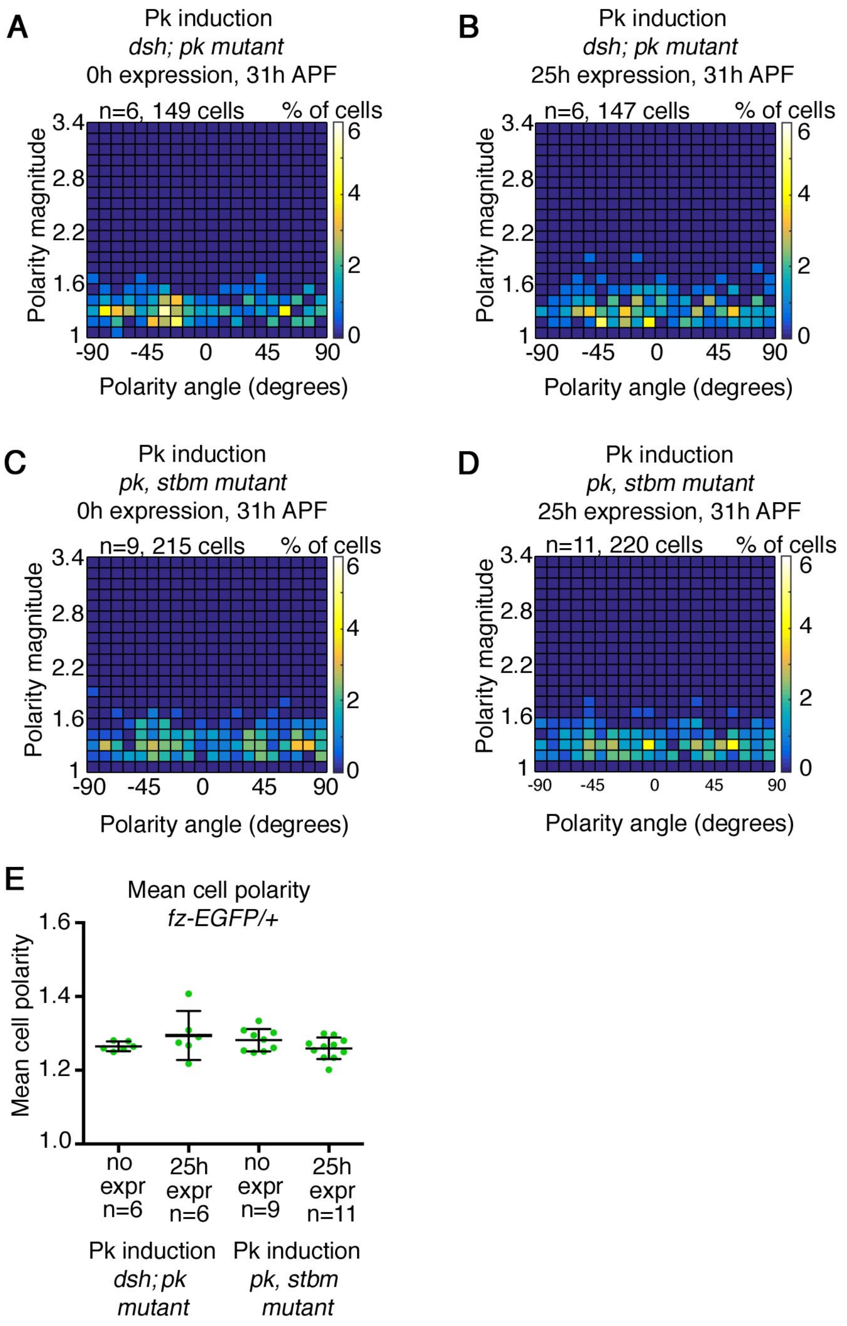

Figure S4. Fz-EGFP polarity after Pk induction in double mutant backgrounds. Related to Figure 5

(A-D) Heat maps of Fz-EGFP in live pupal wings at 31h APF, comparing the angle of polarity against the polarity magnitude, for the induction experiments expressing either Pk (A,B) or Stbm (C,D) for 0h (A,C) or 25h (B,D). Data for the 0h expression wings were collected from the first timepoint of each of the wings used in the FRAP experiments shown in Figure 5A,B. Colours depict the percentage of cells with a particular polarity magnitude and angle. Data has been grouped into 20 bins on each axis.

(E) Scatter plots showing the mean cell polarity of Fz-EGFP in live pupal wings, after induction of Pk for 1h or 25h, in a *stbm*^{6} or *dsh*^{1} mutant background. Data were calculated from the same images as the results in Figure S4A-D. n = number of wings analysed, mean and 95% confidence intervals are shown. t-tests comparing the mean cell polarity of Fz-EGFP: for Pk induction in a *dsh*^{1} mutant comparing 0h vs. 25h P=0.292 and Pk induction in a *stbm*^{6} mutant comparing 0h vs. 25h P=0.115. See Table S1 for full genotypes.

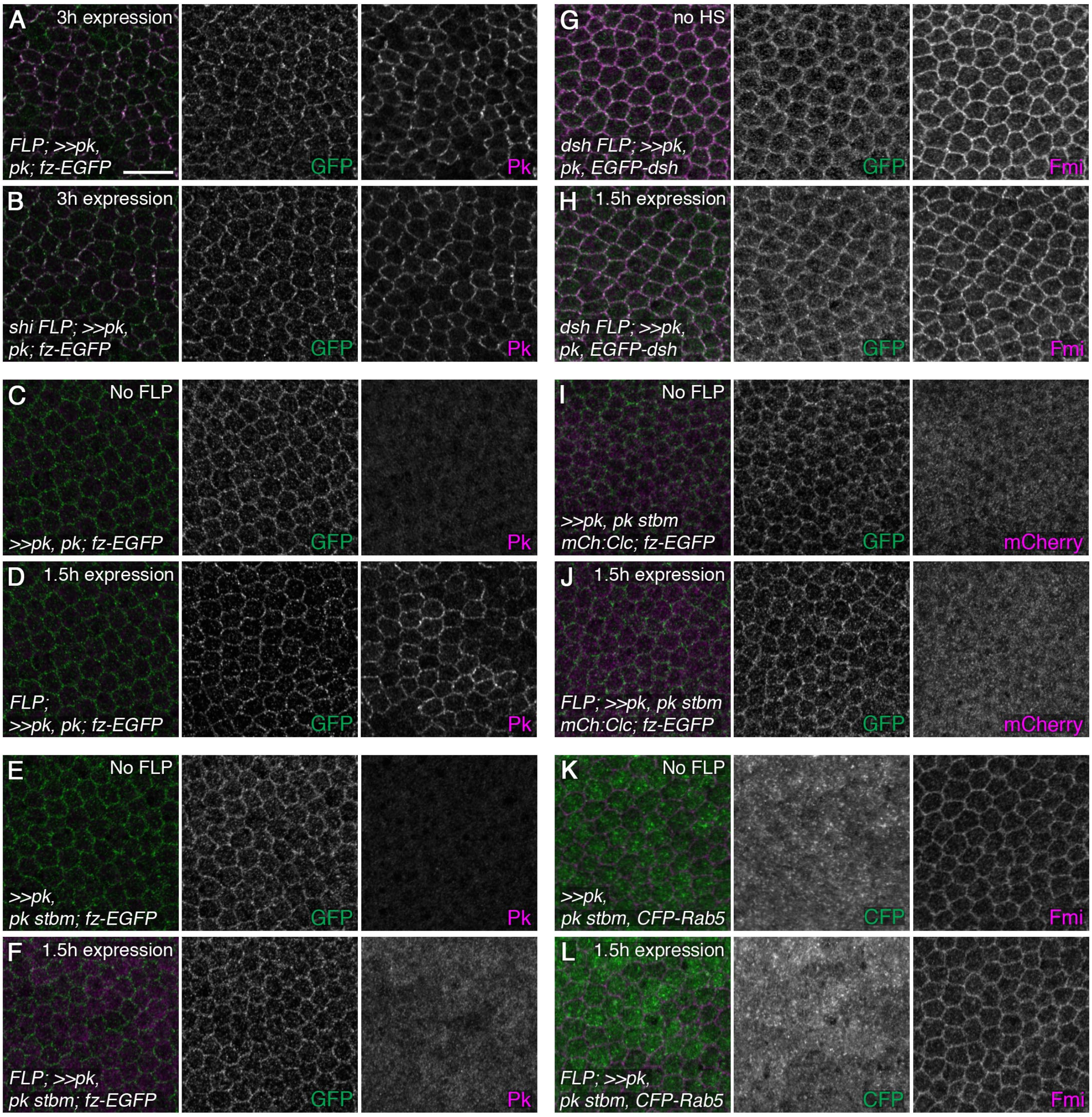

Figure S5. Subcellular localisation of core proteins and endocytic components following Pk induction. Related to Figure 6

(A,B) Images from *hs-FLP; pk*$^{pk-sple-13}$*/Act>>FLAG-pk, pk*$^{pk-sple-13}$*; fz-EGFP/+* (A) or *shi*^{ts1} *FLP12; pk*$^{pk-sple-13}$*/Act>>FLAG-pk, pk*$^{pk-sple-13}$*; fz-EGFP/+* (B) pupal wings, immunolabelled for GFP (green and white) and Pk (magenta and white). Pupae were raised at 18°C for 52h APF, heat shocked for 1h at 38°C to induce Pk expression, and then allowed to develop for a further 3h at the restrictive temperature (29°C). Pk expression is similar in the presence or absence of *shi*. Scale bar 10µm.

(C-F) Images from *pk*$^{pk-sple-13}$*/Act>>FLAG-pk, pk*$^{pk-sple-13}$*; fz-EGFP/+* (C), *hs-FLP; pk*$^{pk-sple-13}$*/Act>>FLAG-pk, pk*$^{pk-sple-13}$*; fz-EGFP/+* (D), *pk*$^{pk-sple-13}$ *stbm*^{6}*/Act>>FLAG-pk, pk*$^{pk-sple-13}$ *stbm*^{6}*; fz-EGFP/+* (E) or *hs-FLP; pk*$^{pk-sple-13}$ *stbm*^{6}*/Act>>FLAG-pk, pk*$^{pk-sple-13}$ *stbm*^{6}*; fz-EGFP/+* (F) pupal wings, immunolabelled for GFP (green and white) and Pk (magenta and white). Pupae were raised at 18°C for 54h APF, heat shocked for 1h at 38°C, and then allowed to develop for a further 1.5h at 29°C. No obvious appearance of Fz-EGFP in vesicles is seen after induction of Pk expression. Note that this temperature regime was chosen, as it shows a rapid destabilisation of Fz-EGFP as assayed by FRAP (see Figure 6B).

(G, H) Images from *dsh*^{1} *FLP12; P[acman]-EGFP-dsh pk*$^{pk-sple-13}$*/Actin>>FLAG-pk, pk*$^{pk-sple-13}$ male prepupae, immunolabelled for GFP (green and white) and Fmi (magenta and white). (G) Pupae were raised at 18°C for 54h APF, and then 1.5h at 29°C. (H) Pupae were raised at 18°C for 54h APF, heat shocked for 1h at 38°C to induce Pk expression, and then allowed to develop for a further 1.5h at 29°C. No obvious appearance of EGFP-Dsh or Fmi in vesicles is seen after induction of Pk expression.

(I-L) *sqh-mCh:Clc, pk*$^{pk\text{-}sple\text{-}13}$ *stbm*^{6}*/Actin>>FLAG-pk, pk*$^{pk\text{-}sple\text{-}13}$ *stbm*^{6}*; fz-EGFP/+* (I), *hs-FLP; sqh-mCh:Clc, pk*$^{pk\text{-}sple\text{-}13}$ *stbm*^{6}*/Actin>>FLAG-pk, pk*$^{pk\text{-}sple\text{-}13}$ *stbm*^{6}*; fz-EGFP/+* (J), *CFP-Rab5, pk*$^{pk\text{-}sple\text{-}13}$ *stbm*^{6}*/Actin>>FLAG-pk, pk*$^{pk\text{-}sple\text{-}13}$ *stbm*^{6}*; fz-EGFP/+* (K) or *hs-FLP; CFP-Rab5, pk*$^{pk\text{-}sple\text{-}13}$ *stbm*^{6}*/Actin>>FLAG-pk, pk*$^{pk\text{-}sple\text{-}13}$ *stbm*^{6}*; fz-EGFP/+* (L) pupal wings, showing GFP immunolabelling (green and white), mCherry fluorescence (magenta and white, I, J) or Fmi immunolabelling (magenta and white, K, L). Pupae were raised at 18°C for 54h APF, heat shocked for 1h at 38°C, and then allowed to develop for a further 1.5h at 29°C. No obvious recruitment of mCh:Clc or CFP-Rab5 to junctions is seen after induction of Pk expression.
